# Supplementary material for: Planar Elongated B12 Structure in M3B12 Clusters (M = Cu-Au)
Source: Molecules. 2022 Dec 28;28(1):236. doi: 10.3390/molecules28010236 (PMC9822480; doi:10.3390/molecules28010236)
Supplement: Supplementary file 1 [file molecules-28-00236-s001.zip › molecules-2085605-supplementary.pdf]

Planar Elongated B_{12} Structure in M_3B_{12} Clusters (M=Cu-Au)

José Solar-Encinas [1], Alejandro Vásquez-Espinal [2,*], Luis Leyva-Parra [1], Osvaldo Yañez [3] , Diego Inostroza [1], Maria Luisa Valenzuela [4], Walter Orellana [5] and William Tiznado [6,*]

[1] Programa de Doctorado en Fisicoquímica Molecular, Facultad de Ciencias Exactas, Universidad Andrés Bello, Av. República 275, Santiago 8370146, Chile

[2] Química y Farmacia, Facultad de Ciencias de la Salud, Universidad Arturo Prat, Casilla 121, Iquique 1100000, Chile

[3] Facultad de Ingeniería y Negocios, Universidad de las Américas, Santiago 7500000, Chile

[4] Grupo de Investigación en Energía y Procesos Sustentables, Instituto de Ciencias Químicas Aplicadas, Facultad de Ingeniería, Universidad Autónoma de Chile, Av. El Llano Subercaseaux 2801, Santiago 8900000, Chile

[5] Departamento de Ciencias Físicas, Universidad Andrés Bello, Santiago 8370136, Chile

[6] Computational and Theoretical Chemistry Group, Departamento de Ciencias Química, Facultad de Ciencias Exactas, Universidad Andrés Bello, Av. República 275, Santiago 8370146, Chile

* Correspondence: alvasquez@unap.cl (A.V.-E.) and wtiznado@unab.cl (W.T.)

SUPPORTING INFORMATION

Figure S1. Global minimum and low-lying isomers of CuB_{12}, their point group symmetries and spectroscopic states. Relative energies are shown in kcal·mol^{-1} at PBE0-D3/def2-TZVP (**in bold**) level including zero-point energy (ZPE) corrections and DLPNO-CCSD(T)/CBS (red parenthesis). A number-letter label identifies structure to facilitate their connection with their Cartesian coordinates (at the end of the ESI).

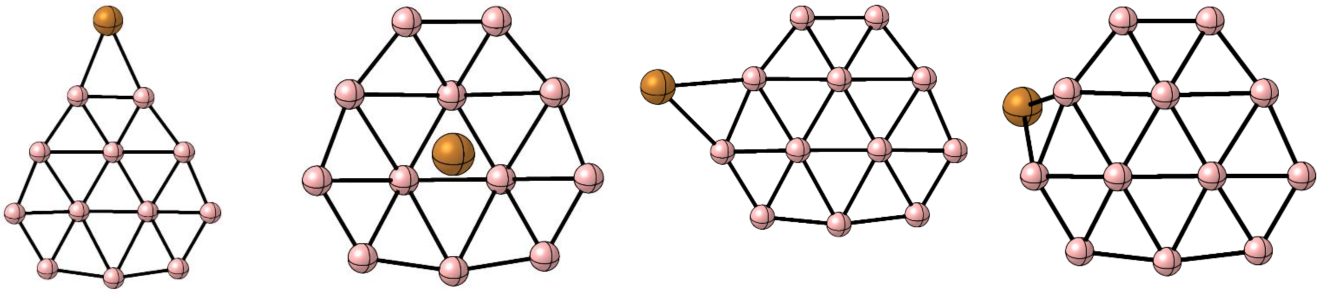

1a (C_s)	1b (C_{3v})	1c (C_1)	1d (C_1)
$^2A'$	$^2A'$	^2A	^2A
**0.0**	**1.7**	**2.3**	**4.7**
(2.8)	(0.0)	(3.3)	(5.1)

Figure S2. Global minimum and low-lying isomers of AgB_{12}, their point group symmetries and spectroscopic states. Relative energies are shown in kcal·mol^{-1} at PBE0-D3/def2-TZVP (**in bold**) level including zero-point energy (ZPE) corrections and DLPNO-CCSD(T)/CBS (red parenthesis). A number-letter label identifies structure to facilitate their connection with their Cartesian coordinates (at the end of the ESI).

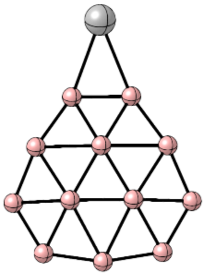

2a (C_s)
$^2A'$
0.0
(0.0)

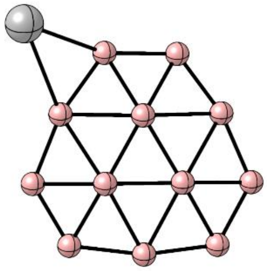

2b (C_1)
^2A
1.5
(1.0)

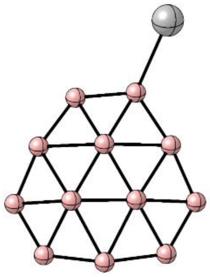

2c (C_1)
^2A
1.8
(3.3)

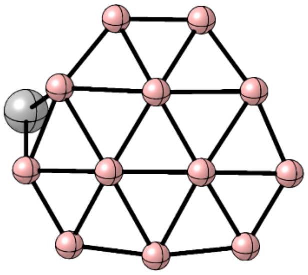

2d (C_1)
^2A
4.1
(4.5)

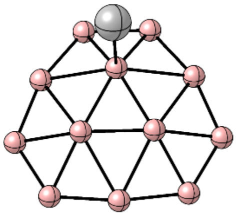

2e (C_s)
$^2A'$
12.3
(11.1)

Figure S3. Global minimum and low-lying isomers of AuB_{12}, their point group symmetries and spectroscopic states. Relative energies are shown in kcal·mol^{-1} at PBE0-D3/def2-TZVP (**in bold**) level including zero-point energy (ZPE) corrections and DLPNO-CCSD(T)/CBS (red parenthesis). A number-letter label identifies structure to facilitate their connection with their Cartesian coordinates (at the end of the ESI).

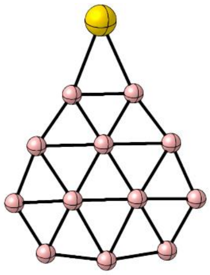

3a (C_s)
$^2A'$
0.0
(0.0)

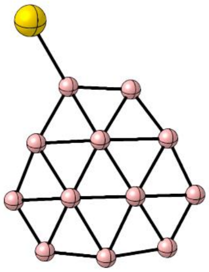

3b (C_1)
^2A
3.8
(4.1)

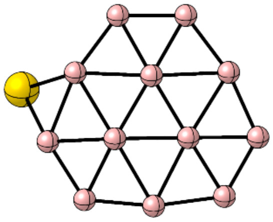

3c (C_1)
^2A
8.0
(7.2)

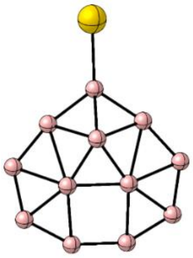

3d (C_s)
$^2A'$
14.3
(13.5)

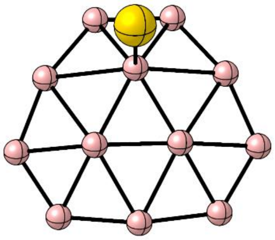

3e (C_s)
$^2A'$
16.4
(14.0)

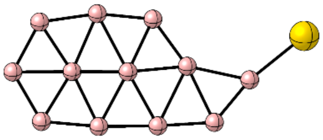

3f (C_1)
^2A
19.8
(18.9)

Figure S4. Global minimum and low-lying isomers of Cu_2B_{12}, their point group symmetries and spectroscopic states. Relative energies are shown in kcal·mol^{-1} at PBE0-D3/def2-TZVP (**in bold**) level including zero-point energy (ZPE) corrections and DLPNO-CCSD(T)/CBS (red parenthesis). A number-letter label identifies structure to facilitate their connection with their Cartesian coordinates (at the end of the ESI).

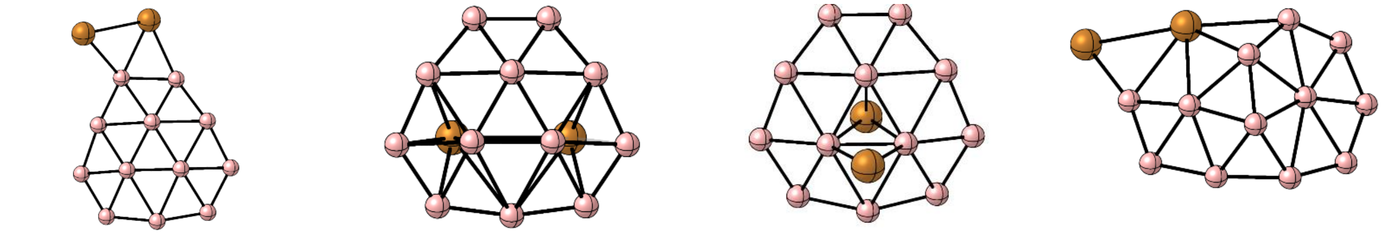

4a (C_1)	4b (C_s)	4c (C_s)	4d (C_1)
^1A	$^1A'$	$^1A'$	^1A
**0.0**	**3.2**	**4.6**	**5.0**
(1.7)	(7.0)	(0.0)	(5.8)

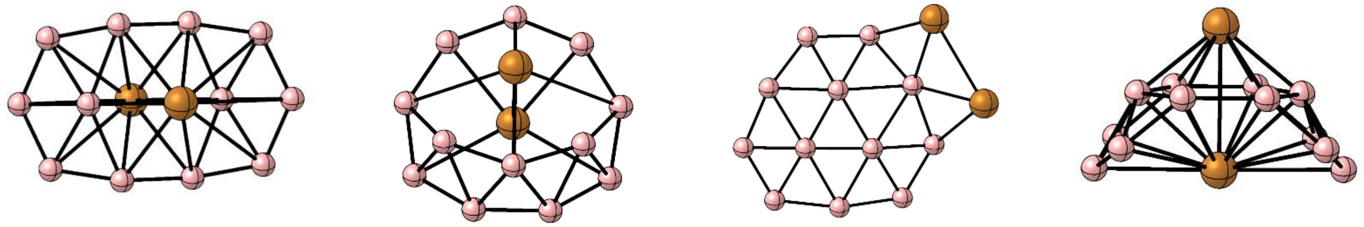

4e (C_{2h})	4f (C_s)	4g (C_1)	4h (C_{2v})
^1A_g	$^1A'$	^1A	$^1A_1$
**5.8**	**6.3**	**6.4**	**6.4**
(1.8)	(6.0)	(9.7)	(3.0)

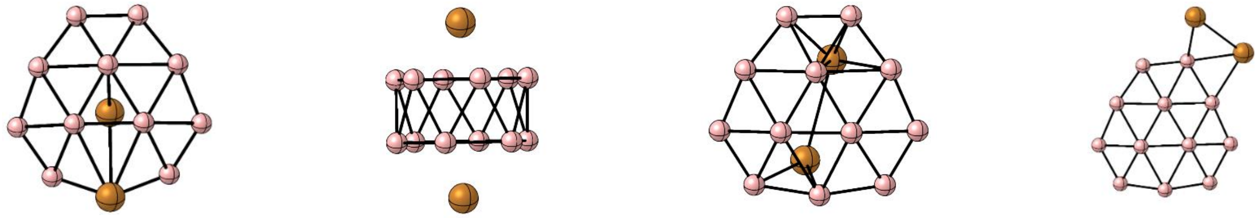

4i (C_s)	4j (D_{6d})	4k (C_1)	4l (C_1)
$^1A'$	$^1A_1$	^1A	^1A
**7.4**	**7.6**	**7.8**	**7.9**
(3.7)	(7.6)	(11.9)	(9.3)

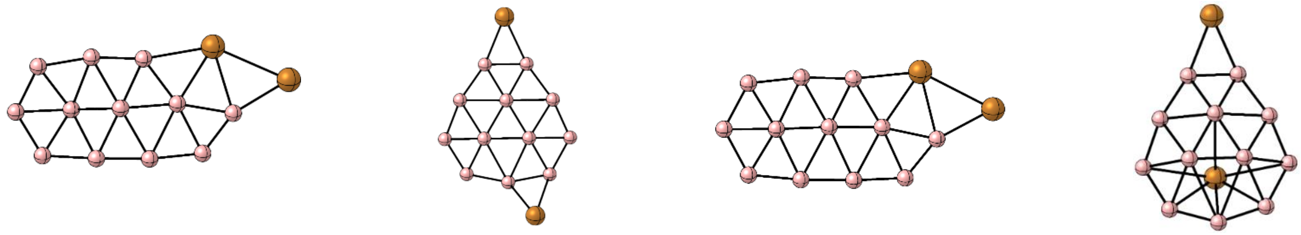

4m (C_1)	4n (C_1)	4o (C_1)	4p (C_s)
^1A	^1A	^3A	$^1A'$
**8.8**	**9.2**	**10.7**	**10.8**
(10.8)	(8.4)		

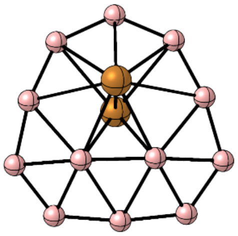

4q (C_s)
$^1A'$
11.1

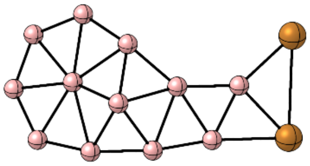

4r (C_1)
^1A
12.0

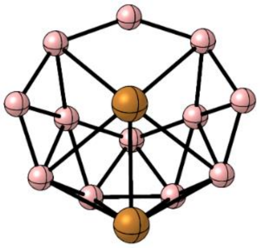

4s (C_s)
$^1A'$
13.3

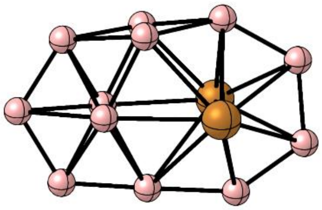

4t (C_s)
$^1A'$
14.1

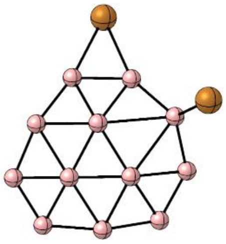

4u (C_1)
^1A
14.3

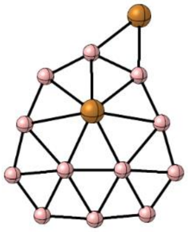

4v (C_1)
^1A
15.4

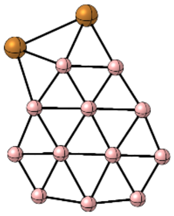

4w (C_1)
^3A
15.5

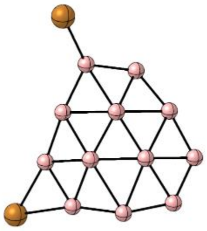

4x (C_1)
^1A
16.1

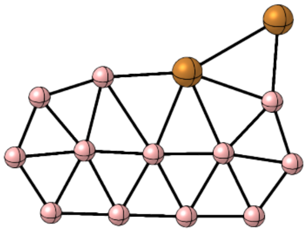

4y (C_s)
$^1A'$
17.8

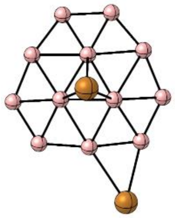

4z (C_1)
^1A
18.6

Figure S5. Global minimum and low-lying isomers of Ag_2B_{12}, their point group symmetries and spectroscopic states. Relative energies are shown in kcal·mol^{-1} at PBE0-D3/def2-TZVP (**in bold**) level including zero-point energy (ZPE) corrections and DLPNO-CCSD(T)/CBS (red parenthesis). A number-letter label identifies structure to facilitate their connection with their Cartesian coordinates (at the end of the ESI).

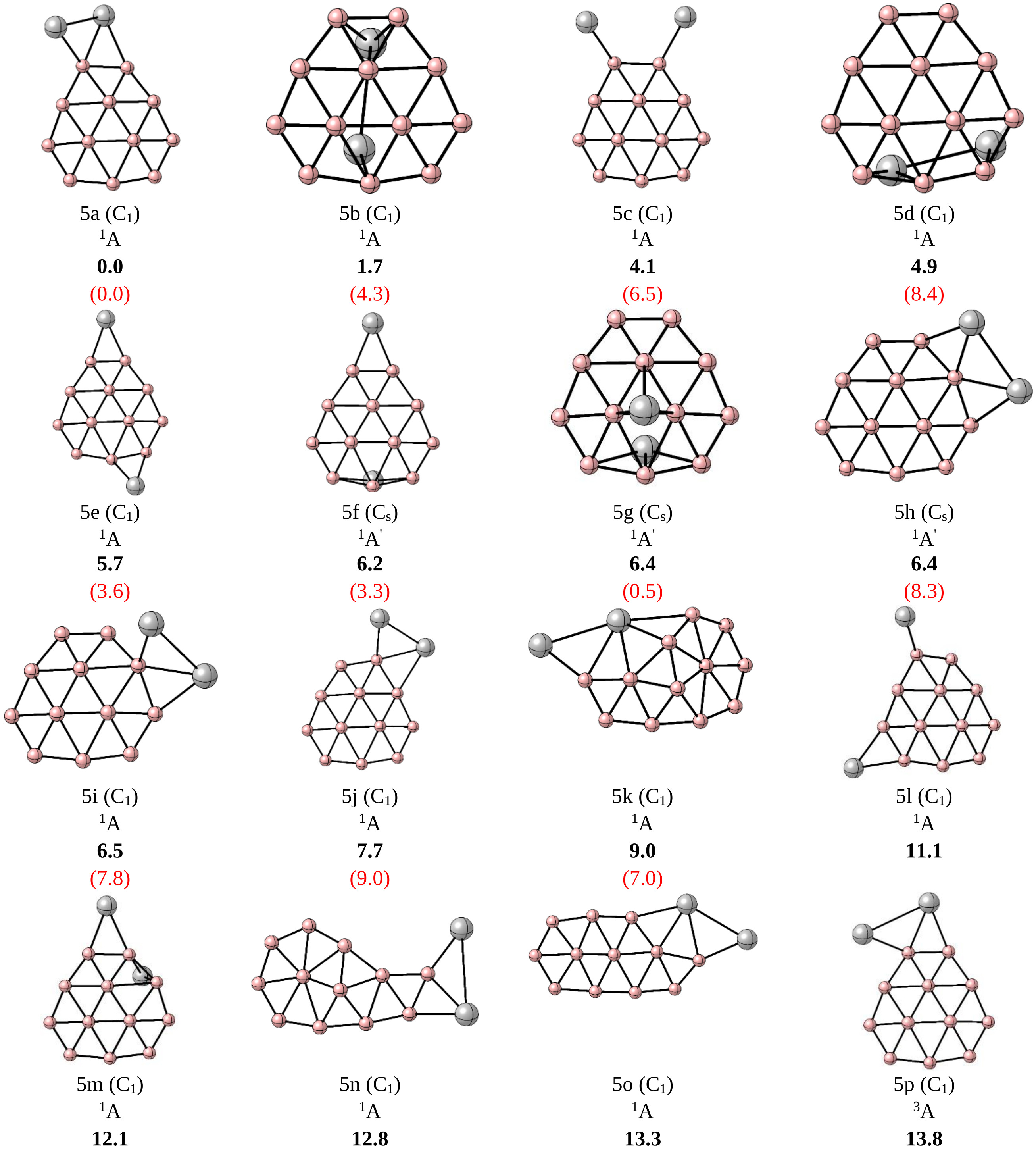

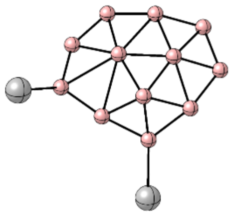

5q (C_1)
^1A
14.2

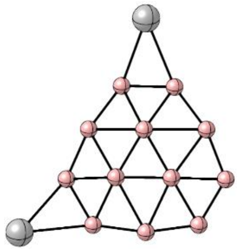

5r (C_1)
^3A
14.3

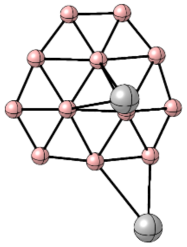

5s (C_1)
^1A
14.5

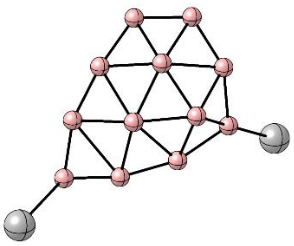

5t (C_1)
^1A
15.2

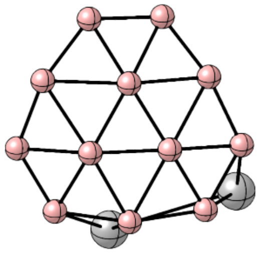

5u (C_1)
^3A
15.7

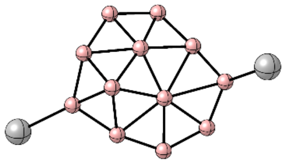

5v (C_1)
^1A
16.3

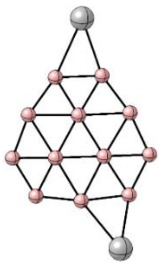

5w (C_1)
^3A
16.7

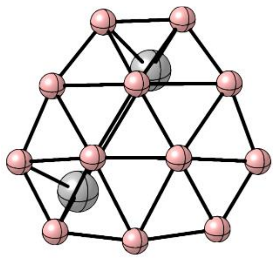

5x (C_s)
$^3A'$
17.3

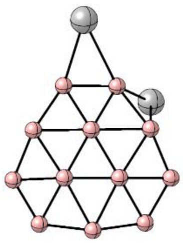

5y (C_1)
^3A
18.0

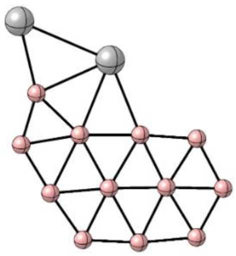

5z (C_s)
$^3A'$
18.3

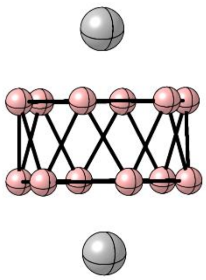

5a¨ (D_{6d})
$^1A_1$
54.7

Figure S6. Global minimum and low-lying isomers of Au_2B_{12}, their point group symmetries and spectroscopic states. Relative energies are shown in kcal·mol^{-1} at PBE0-D3/def2-TZVP (**in bold**) level including zero-point energy (ZPE) corrections and DLPNO-CCSD(T)/CBS (red parenthesis). A number-letter label identifies structure to facilitate their connection with their Cartesian coordinates (at the end of the ESI).

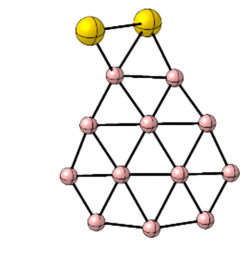

6a (C_1)
^1A
0.0
(0.0)

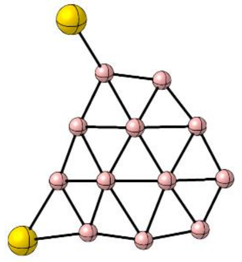

6b (C_1)
^1A
1.2
(11.2)

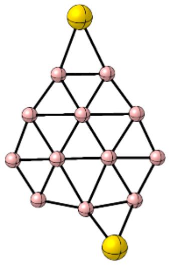

6c (C_1)
^1A
9.6
(7.7)

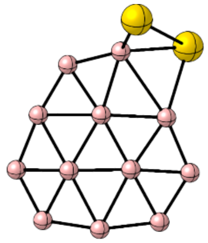

6d (C_1)
^1A
10.6
(9.0)

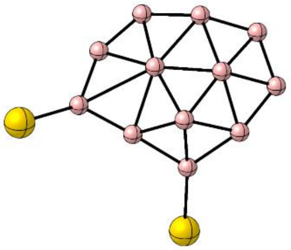

6e (C_1)
^1A
11.4
(9.6)

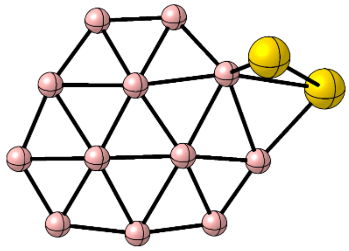

6f (C_1)
^1A
12.0
(10.6)

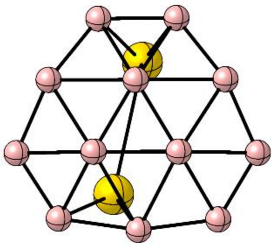

6g (C_1)
^1A
12.2
(13.0)

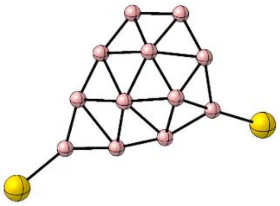

6h (C_1)
^1A
12.4
(12.1)

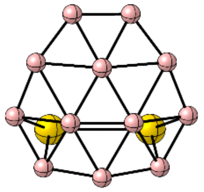

6i (C_1)
^1A
12.5

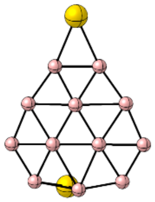

6j (C_1)
^1A
12.6

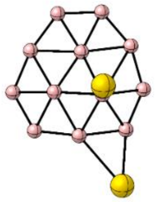

6k (C_1)
^1A
14.4

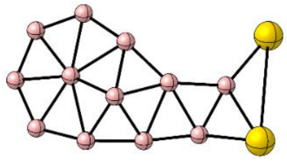

6l (C_1)
^1A
14.9

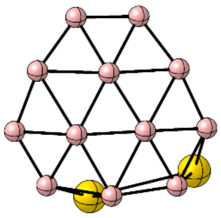

6m (C_1)
^1A
15.2

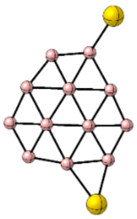

6n (C_1)
^1A
15.5

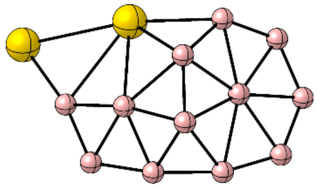

6o (C_1)
^1A
16.7

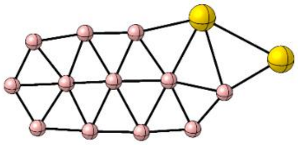

6p (C_1)
^1A
17.7

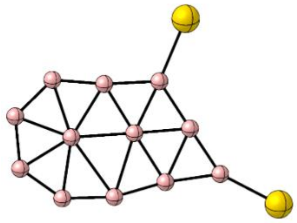

6q (C_s)
$^1A'$
17.8

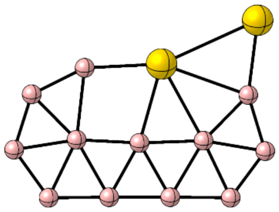

6r (C_s)
$^1A'$
18.3

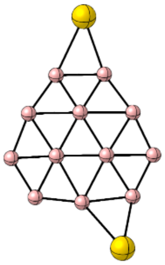

6s (C_1)
^3A
19.8

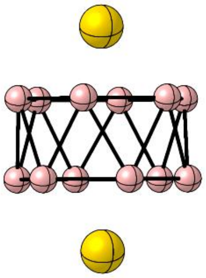

6t (D_{6d})
$^1A_1$
102.6

Figures S7. Global minimum and low-lying isomers of Cu_3B_{12}, their point group symmetries and spectroscopic states. Relative energies are shown in kcal·mol^{-1} at PBE0-D3/def2-TZVP (**in bold**) level including zero-point energy (ZPE) corrections and DLPNO-CCSD(T)/CBS (red parenthesis). A number-letter label identifies structure to facilitate their connection with their Cartesian coordinates (at the end of the ESI).

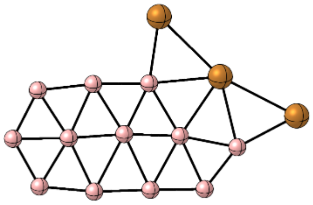

7a (C_s)
$^2A''$
0.0
(0.0)

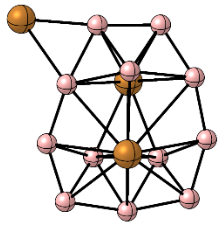

7b (C_1)
^2A
6.3
(1.2)

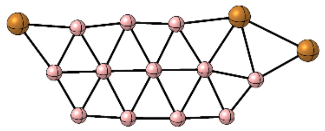

7c (C_s)
$^2A''$
8.3
(12.2)

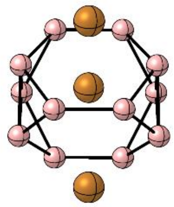

7d (C_s)
$^2A'$
9.0

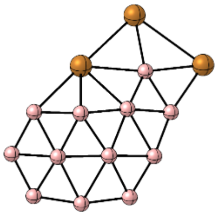

7e (C_1)
^2A
11.7

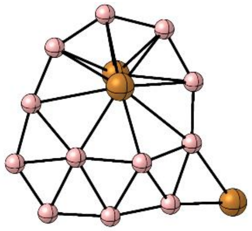

7f (C_1)
^2A
14.8

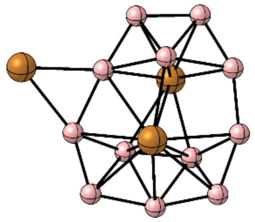

7g (C_1)
^2A
15.9

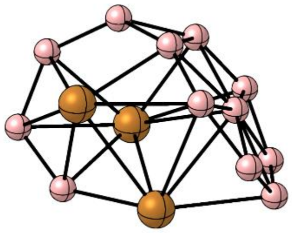

7h (C_1)
^2A
16.4

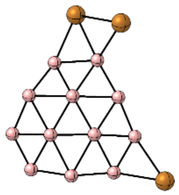

7i (C_1)
^2A
16.9

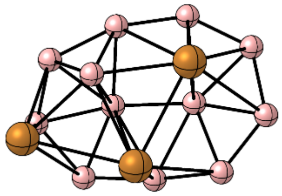

7j (C_1)
^2A
18.2

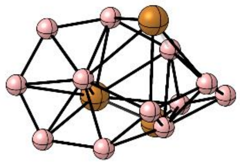

7k (C_1)
^2A
19.3

Figure S8. Global minimum and low-lying isomers of Ag_3B_{12}, their point group symmetries and spectroscopic states. Relative energies are shown in kcal·mol^{-1} at PBE0-D3/def2-TZVP (**in bold**) level including zero-point energy (ZPE) corrections and DLPNO-CCSD(T)/CBS (red parenthesis). A number-letter label identifies structure to facilitate their connection with their Cartesian coordinates (at the end of the ESI).

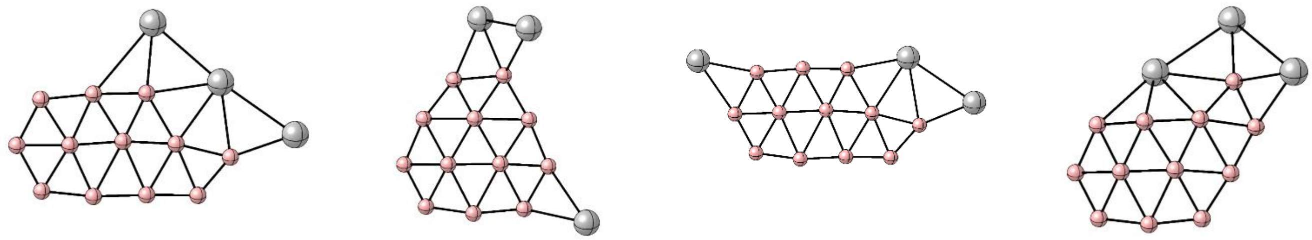

8a (C_s)	8b (C_1)	8c (C_s)	8d (C_1)
$^2A'$	^2A	$^2A''$	^2A
**0.0**	**10.7**	**10.7**	**11.9**
(0.0)	(10.3)	(15.4)	

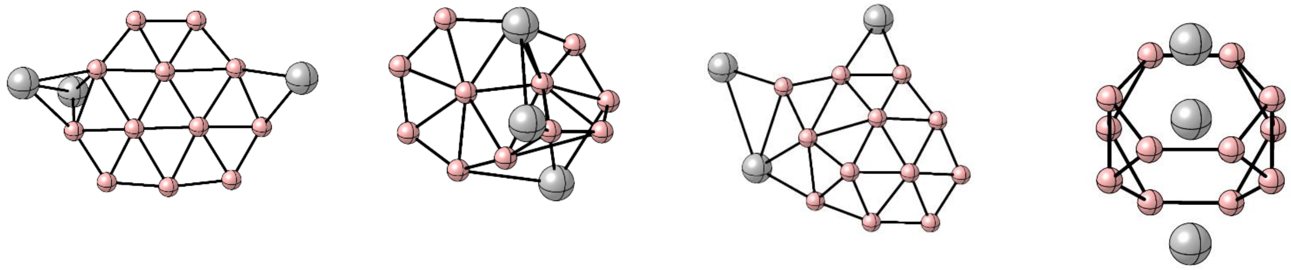

8e (C_1)	8f (C_1)	8g (C_1)	8h (C_s)
^2A	^2A	^2A	$^2A'$
**15.1**	**16.5**	**19.9**	**62.6**

Figure S9. Global minimum and low-lying isomers of Au_3B_{12}, their point group symmetries and spectroscopic states. Relative energies are shown in kcal·mol^{-1} at PBE0-D3/def2-TZVP (**in bold**) level including zero-point energy (ZPE) corrections and DLPNO-CCSD(T)/CBS (red parenthesis). A number-letter label identifies structure to facilitate their connection with their Cartesian coordinates (at the end of the ESI).

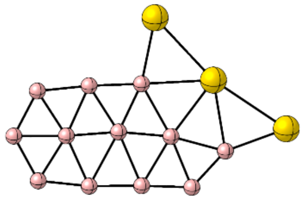

9a (C_s)
$^2A''$
0.0
(0.0)

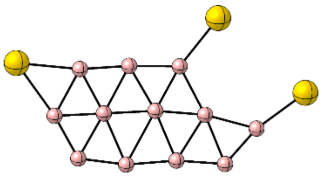

9b (C_s)
$^2A''$
1.9
(0.9)

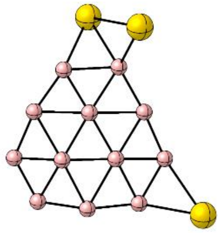

9c (C_1)
^2A
2.1
(0.4)

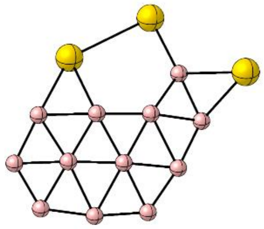

9d (C_1)
^2A
5.6
(7.3)

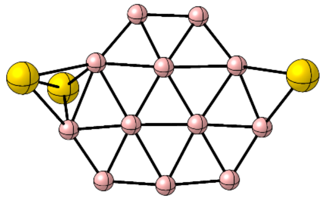

9e (C_1)
^2A
8.2
(8.6)

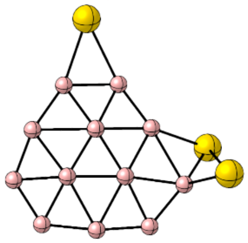

9f (C_1)
^2A
9.7

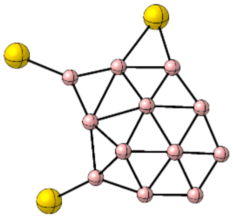

9g (C_1)
^2A
10.7

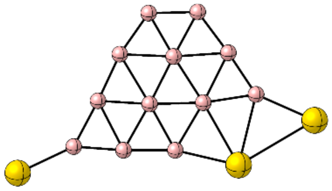

9h (C_s)
$^2A'$
15.7

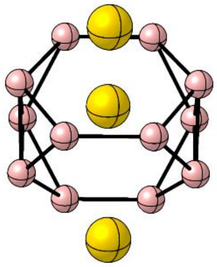

9i (C_s)
$^2A'$
112.2

Figure S10. Bond lengths (r, Å), natural charges (q, |e|), and Wiberg bond indices (WBI) of the CuB_{12}, AgB_{12} and AuB_{12} GMs at the PBE0-D3/def2-TZVP level.

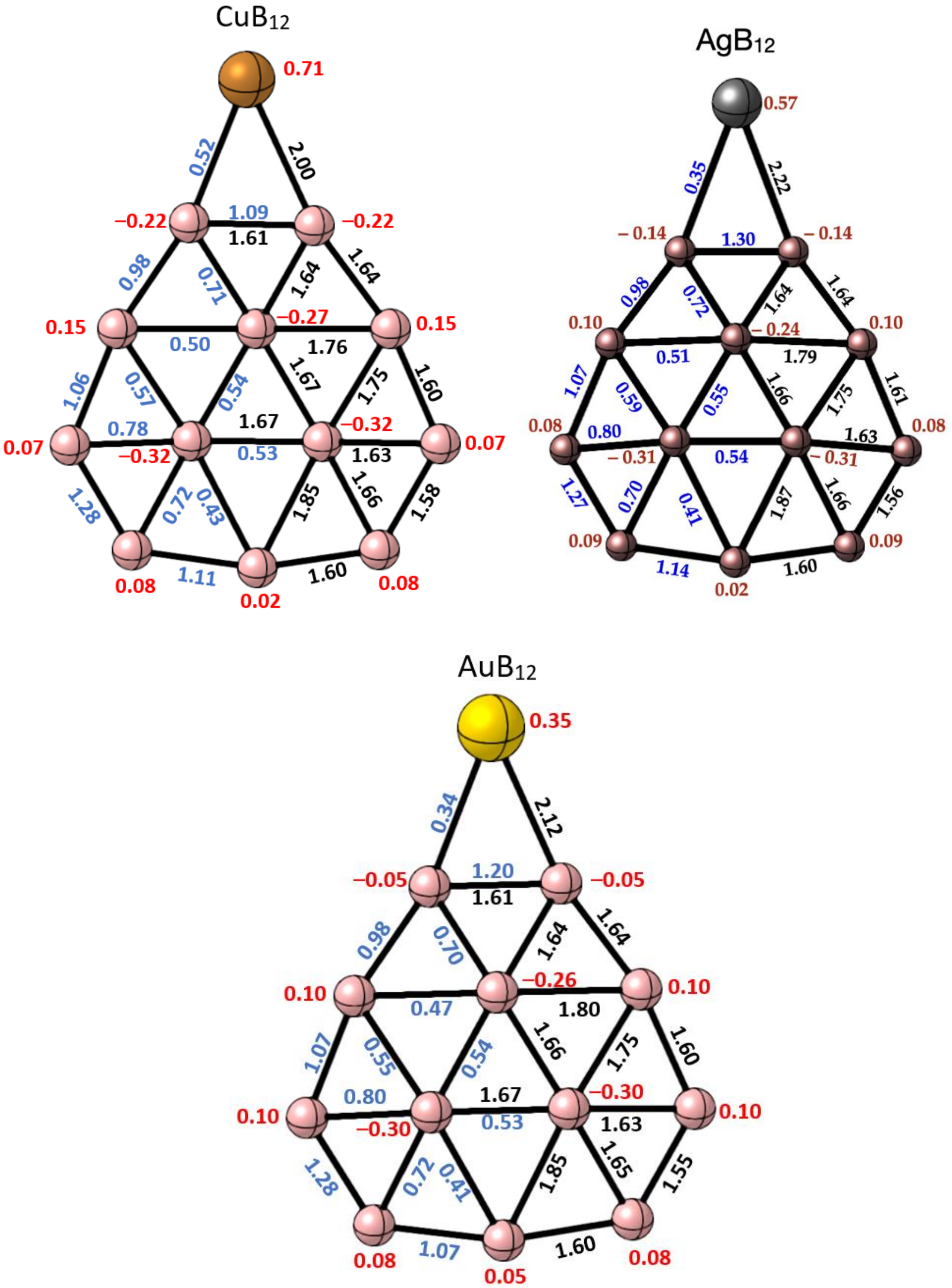

Figure S11. Bond lengths (r, Å), natural charges (q, |e|), and **Wiberg bond indices (WBI)** of the Cu_2B_{12}, Ag_2B_{12} and Au_2B_{12} GMs at the PBE0-D3/def2-TZVP level.

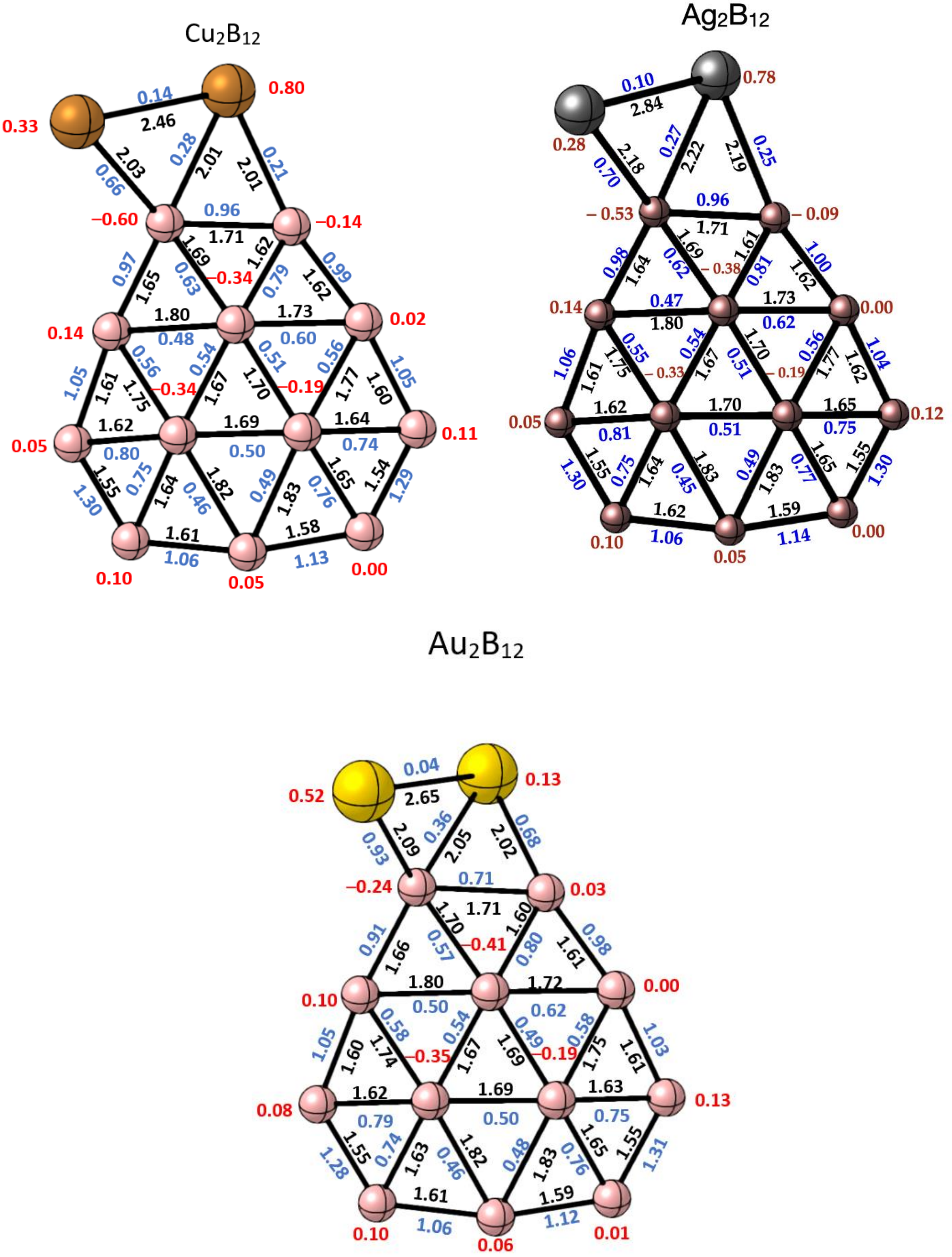

Figure S12. Bond lengths (r, Å), natural charges (q, |e|), and Wiberg bond indices (WBI) of the Cu_3B_{12}, Ag_3B_{12} and Au_3B_{12} GMs at the PBE0-D3/def2-TZVP level.

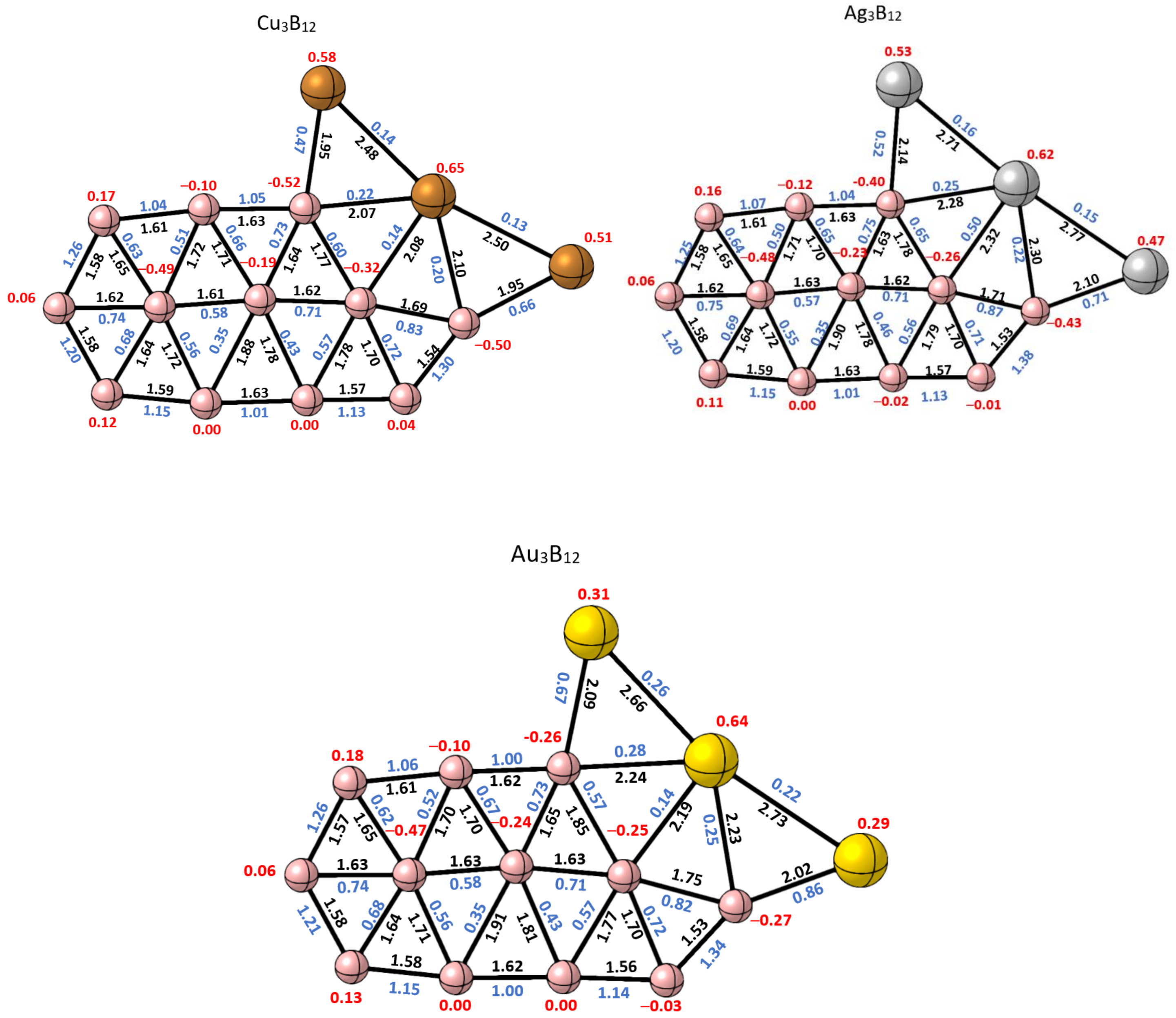

Figure S13. The AdNDP bonding pattern of the CuB_{12}, AgB_{12} and AuB_{12} GMs at the PBE0-D3/def2-TZVP level.

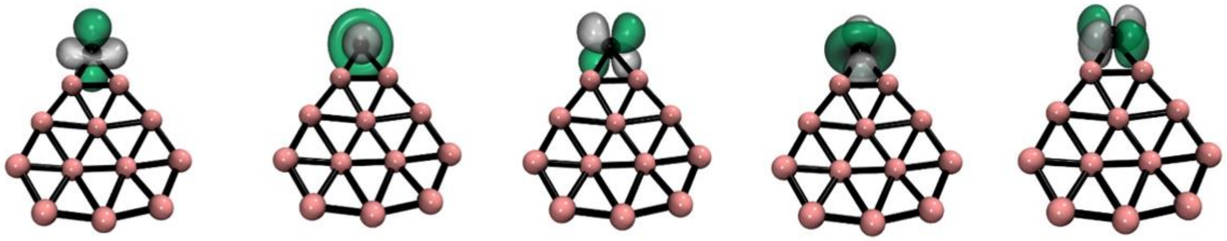

5 x 1c-2e M LPs

**M = Cu**	ON = 1.99 – 1.90 |e|
**M = Ag**	ON = 1.99 – 1.90 |e|
**M = Au**	ON = 1.99 – 1.80 |e|

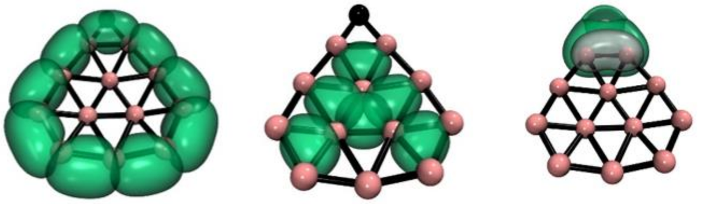

9 x 2c-2e σ-bonds | 6 x 3c-2e σ-bonds | 1 x 3c-1e σ-bond

	9 x 2c-2e σ-bonds	6 x 3c-2e σ-bonds	1 x 3c-1e σ-bond
**M = Cu**	ON = 1.93 - 1.80 |e|	ON = 1.90 – 1.79 |e|	ON = 0.89 |e|
**M = Ag**	ON = 1.93 - 1.81 |e|	ON = 1.91 – 1.79 |e|	ON = 0.91 |e|
**M = Au**	ON = 1.93 - 1.71 |e|	ON = 1.90 – 1.79 |e|	ON = 0.95 |e|

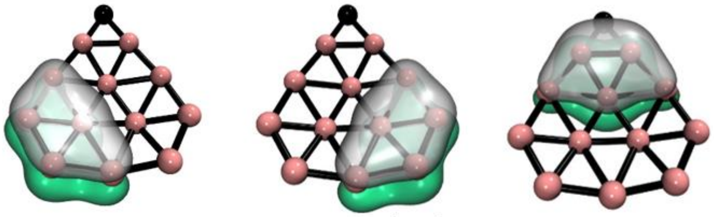

3 x 5c-2e π-bonds

**M = Cu**	ON = 1.88 – 1.85 |e|
**M = Ag**	ON = 1.85 – 1.71 |e|
**M = Au**	ON = 1.86 – 1.78 |e|

Figure S14. The AdNDP bonding pattern of the Cu_2B_{12}, Ag_2B_{12} and Au_2B_{12} GMs at the PBE0-D3/def2-TZVP level.

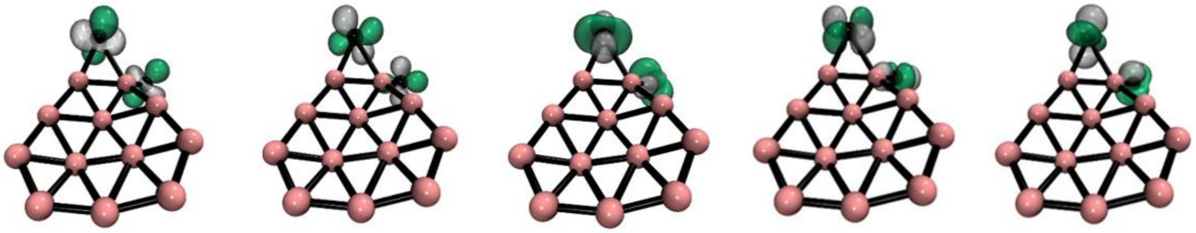

10 x 1c-2e M LPs

**M = Cu**	ON = 1.99 – 1.92 |e|
**M = Ag**	ON = 1.99 – 1.90 |e|
**M = Au**	ON = 1.99 – 1.87 |e|

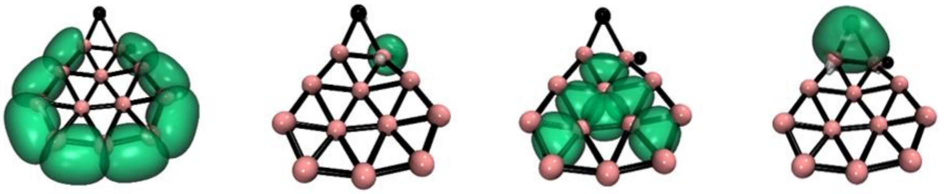

	8 x 2c-2e σ-bonds	1 x 2c-2e σ-bond	6 x 3c-2e σ-bonds	1 x 3c-2e σ-bond
**M = Cu**	ON = 1.93 - 1.86 |e|	ON = 1.78 |e|	ON = 1.93 – 1.81 |e|	ON = 1.90 |e|
**M = Ag**	ON = 1.93 - 1.86 |e|	ON = 1.80 |e|	ON = 1.89 – 1.81 |e|	ON = 1.92 |e|
**M = Au**	ON = 1.93 - 1.87 |e|	ON = 1.93 |e|	ON = 1.95 – 1.80 |e|	ON = 1.95 |e|

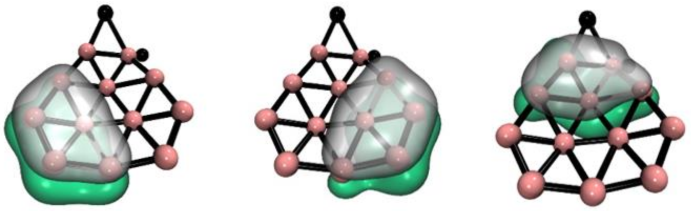

3 x 5c-2e π-bonds

**M = Cu**	ON = 1.89 – 1.76 |e|
**M = Ag**	ON = 1.88 – 1.78 |e|
**M = Au**	ON = 1.88 – 1.74 |e|

Figure S15. The AdNDP bonding pattern of the Cu_3B_{12}, Ag_3B_{12} and Au_3B_{12} GMs at the PBE0-D3/def2-TZVP level.

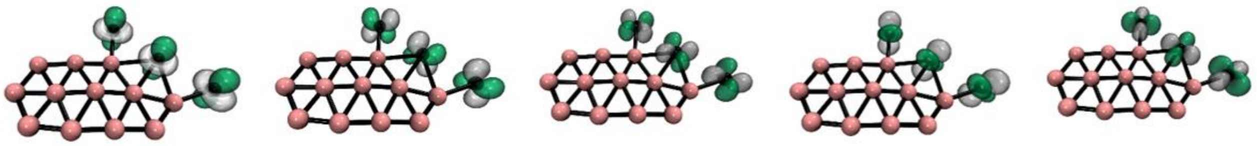

	15 x 1c-2e M LPs
**M = Cu**	ON = 1.99 – 1.93 |e|
**M = Ag**	ON = 1.99 – 1.93 |e|
**M = Au**	ON = 1.99 – 1.87 |e|

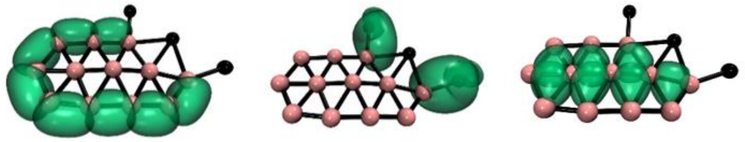

	8 x 2c-2e σ-bonds	2 x 2c-2e σ-bonds	7 x 3c-2e σ-bonds
**M = Cu**	ON = 1.94 – 1.82 |e|	ON = 1.79 – 1.76 |e|	ON = 1.94 – 1.78 |e|
**M = Ag**	ON = 1.94 – 1.71 |e|	ON = 1.79 – 1.77 |e|	ON = 1.94 – 1.78 |e|
**M = Au**	ON = 1.94 – 1.70 |e|	ON = 1.82 – 1.71 |e|	ON = 1.91 – 1.77 |e|

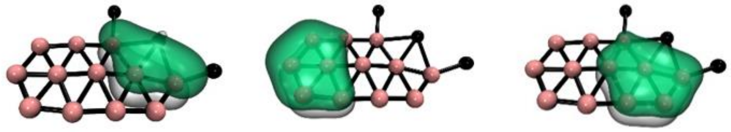

	1 x 4c-1e π-bond	2 x 5c-2e π-bonds
**M = Cu**	ON = 0.88 |e|	ON = 1.91 – 1.89 |e|
**M = Ag**	ON = 0.92 |e|	ON = 1.93 – 1.92 |e|
**M = Au**	ON = 0.98 |e|	ON = 1.93 – 1.92 |e|

Figure S16. Energy cycle for the isomerization between the GM (top) and the higher energy isomer (bottom) of M_3B_{12} (M = Cu, Ag, Au) used in IEDA computations.

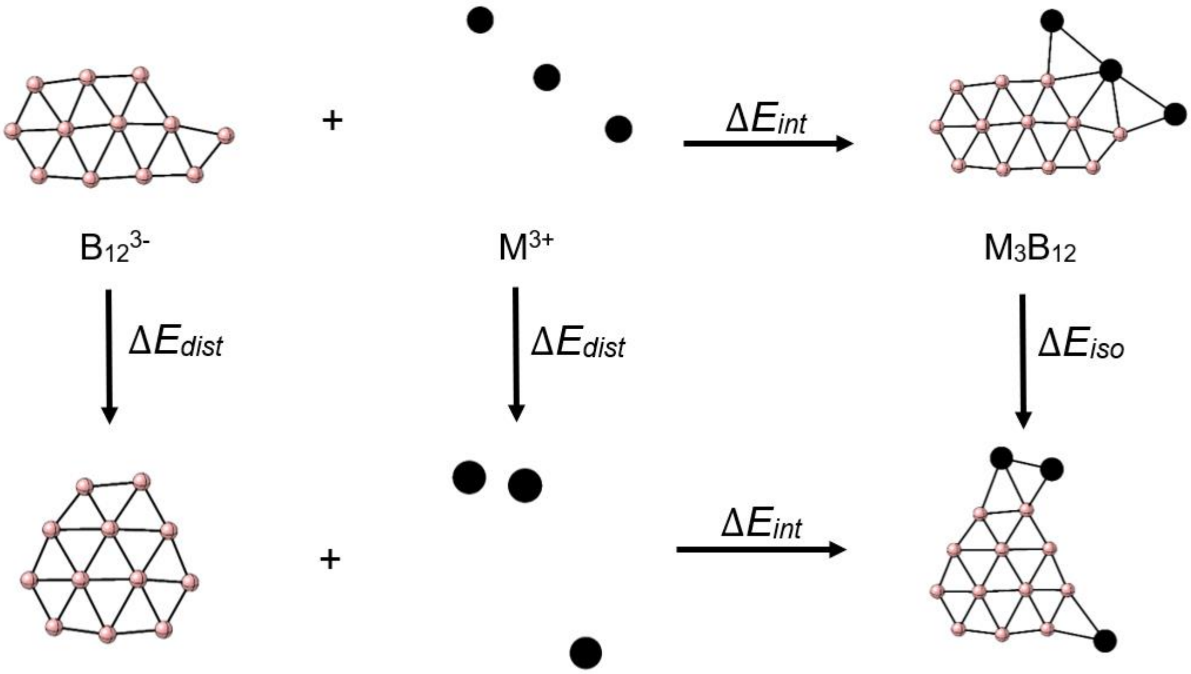

Figure S17. The susceptibility of density current for Cu_3B_{12} in a plane 0.5 Å above the molecular plane (vector plot). The scheme for identifying different ring current circuits through a computed profile of the ring current strength (RCS) passing selected planes. The RCS profiles and the identified ring current circuits with their respective RCS value in $nA.T^{-1}$

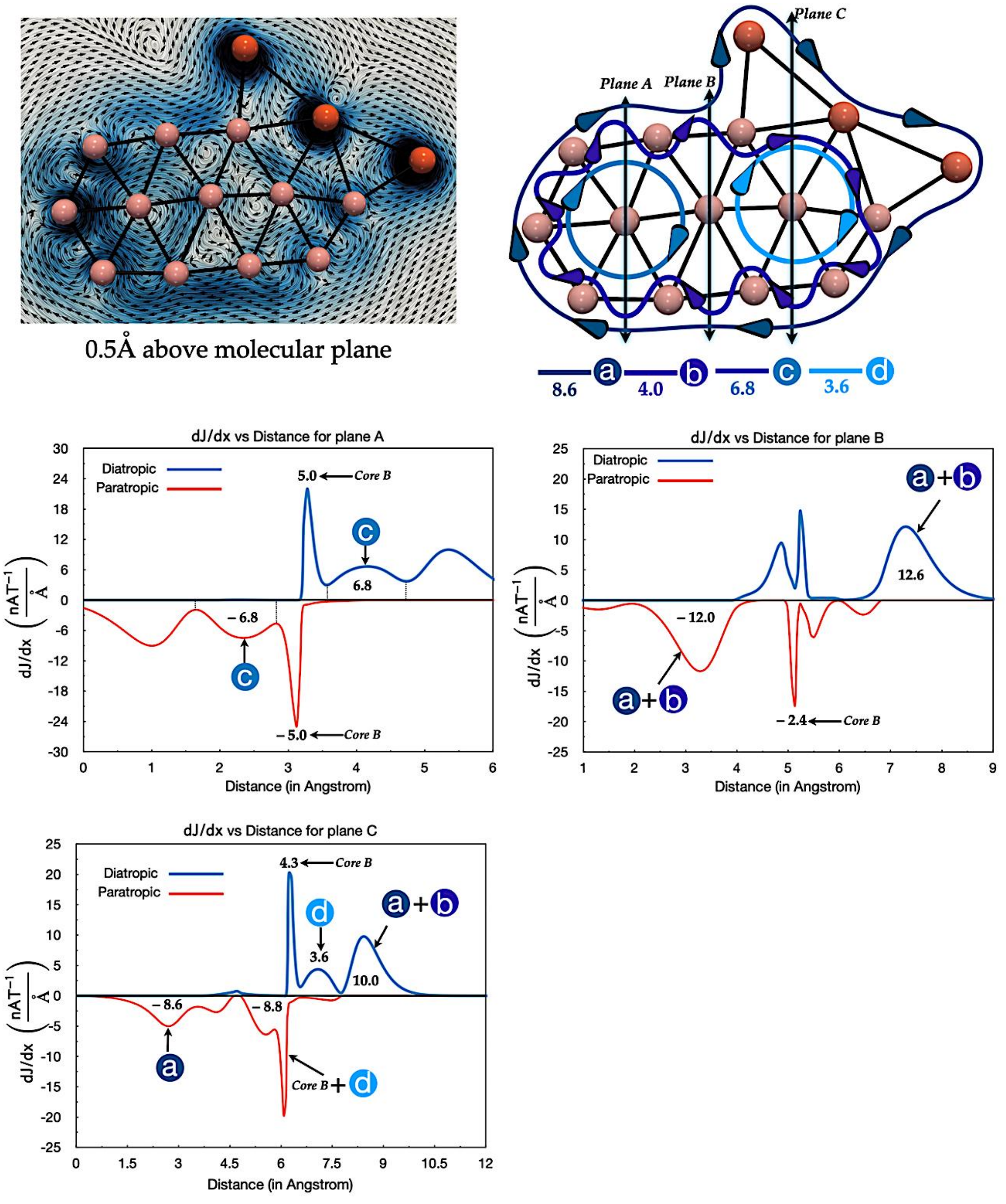

Figure S18. The susceptibility of density current for Ag_3B_{12} in a plane 0.5 Å above the molecular plane (vector plot). The scheme for identifying different ring current circuits through a computed profile of the ring current strength (RCS) passing selected planes. The RCS profiles and the identified ring current circuits with their respective RCS value in $nA.T^{-1}$

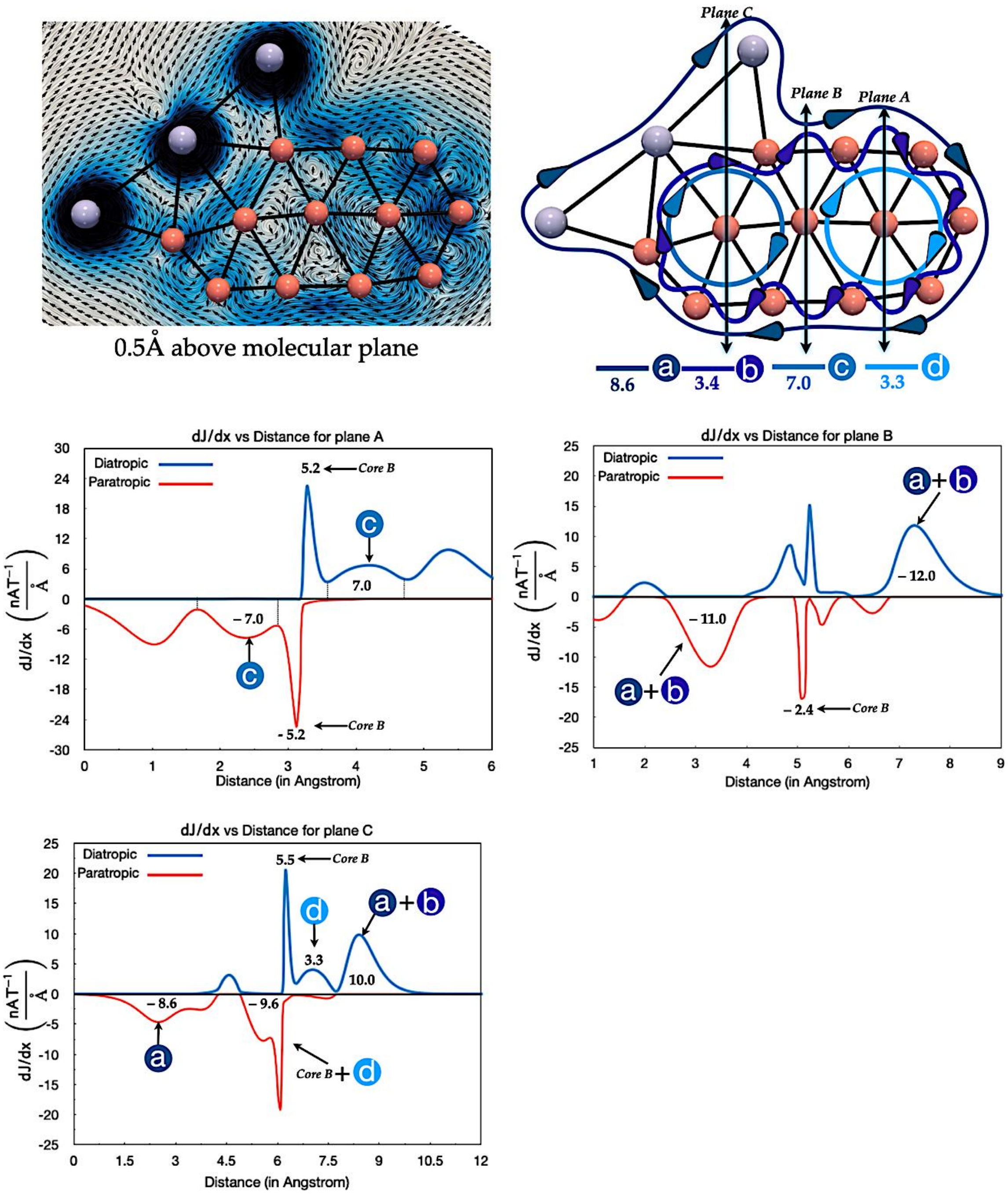

Figure S19. The susceptibility of density current for Au_3B_{12} in a plane 0.5 Å above the molecular plane (vector plot). The scheme for identifying different ring current circuits through a computed profile of the ring current strength (RCS) passing selected planes. The RCS profiles and the identified ring current circuits with their respective RCS value in $nA.T^{-1}$

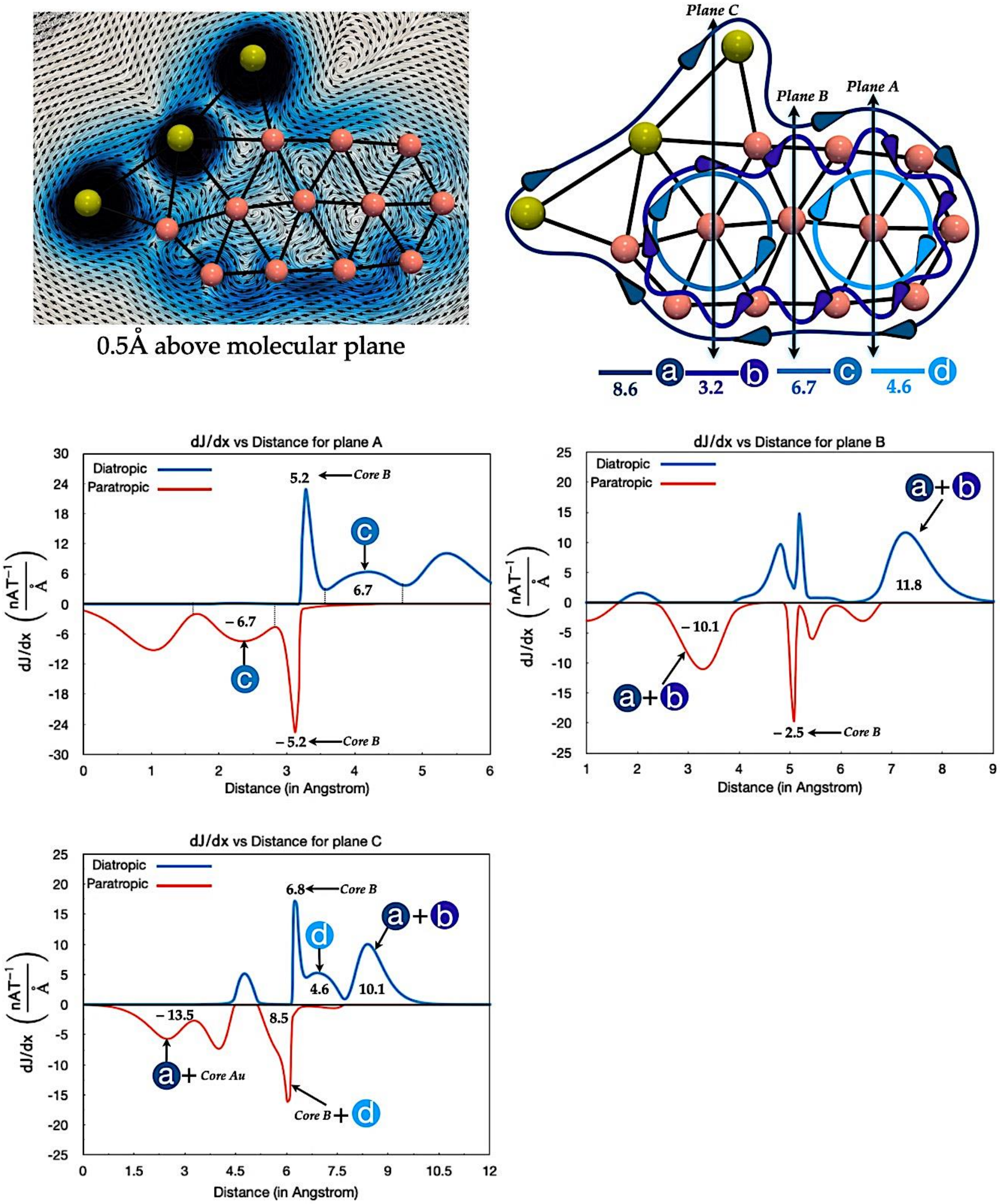

Table S1. Computed natural electron configurations of the Cu, Ag and Au atoms in the putative global minima of MB_{12}, M_2B_{12} and M_3B_{12} systems

	MB_{12}	M_2B_{12}	M_3B_{12}
Cu	[Ar]$3d^{9.85}4s^{0.42}4p^{0.01}$	[Ar] $3d^{9.87}4s^{0.31}4p^{0.02}4d^{0.01}$<br>[Ar]$3d^{9.89}4s^{0.76}4p^{0.01}$	[Ar]$3d^{9.89}4s^{0.59}4p^{0.01}$<br>[Ar]$3d^{9.90}4s^{0.51}4p^{0.01}$<br>[Ar]$3d^{9.85}4s^{0.47}4p^{0.01}4d^{0.01}$
Ag	[Kr]$4d^{9.86}5s^{0.56}$ $5p^{0.01}$	[Kr]$4d^{9.90}5s^{0.81}$<br>[Kr]$4d^{9.88}5s^{0.31}5p^{0.02}$	[Kr]$4d^{9.90}5s^{0.62}5p^{0.01}$<br>[Kr]$4d^{9.90}5s^{0.55}5p^{0.01}$<br>[Kr]$4d^{9.86}5s^{0.49}5p^{0.01}5d^{0.01}$
Au	[Xe]$4f^{14}5d^{9.67}6s^{0.94}6p^{0.01}7s^{0.01}6d^{0.01}$	[Xe]$4f^{14}5d^{9.77}6s^{1.09}$<br>[Xe]$4f^{14}5d^{9.73}6s^{0.74}$	[Xe]$4f^{14}5d^{9.77}6s^{0.92}$<br>[Xe]$4f^{14}5d^{9.80}6s^{0.88}$<br>[Xe]$4f^{14}5d^{9.72}6s^{0.61}6p^{0.01}6d^{0.02}$

Cartesian Coordinates

Cartesian Coordinates of isomers of the MnB12 (M = Cu, Ag, Au) (n = 1-3) clusters at the PBE0-D3/def2-TZVP level.

**1a**				**1b**			
B	-1.829609000	-2.358315000	-0.198053000	B	-1.757733000	1.014828000	-0.362160000
B	-1.863318000	2.370056000	-0.182873000	Cu	0.000000000	0.000000000	0.924454000
B	-3.177924000	-1.583553000	-0.120911000	B	-0.767479000	2.240394000	-0.180225000
B	-3.206498000	1.576512000	-0.127826000	B	1.556499000	-1.784854000	-0.180225000
B	-0.347647000	-1.741961000	-0.102994000	B	1.757733000	1.014828000	-0.362160000
Cu	2.821052000	-0.054865000	-0.112361000	B	0.000000000	-2.029656000	-0.362160000
B	-1.788559000	-0.828200000	0.337157000	B	0.838774000	-0.484266000	-1.064666000
B	-0.386539000	1.749573000	-0.069989000	B	0.000000000	0.968533000	-1.064666000
B	-3.420116000	0.009697000	0.022450000	B	-0.838774000	-0.484266000	-1.064666000
B	-1.813665000	0.849386000	0.413797000	B	-1.556499000	-1.784854000	-0.180225000
B	0.997713000	-0.808934000	-0.108699000	B	-2.323978000	-0.455541000	-0.180225000
B	0.934685000	0.793945000	-0.277256000	B	2.323978000	-0.455541000	-0.180225000
B	-0.357535000	0.021571000	0.304096000	B	0.767479000	2.240394000	-0.180225000
**1c**				**1d**			
B	-1.063395000	0.948857000	-0.315927000	B	-0.704946000	0.678795000	0.918379000
B	-3.721650000	-0.060628000	-0.100649000	B	2.840382000	-1.517559000	-0.617391000
Cu	2.589385000	0.128130000	-0.150207000	Cu	-2.239593000	0.210760000	-0.380489000
B	-2.823101000	1.286626000	-0.094461000	B	3.329572000	-0.066418000	-0.822438000
B	-0.491290000	-0.637591000	-0.183550000	B	-0.069300000	2.102516000	0.481008000
B	-0.059352000	-2.115866000	0.352499000	B	1.431231000	-1.993496000	0.073692000
B	-0.144748000	2.099067000	0.398256000	B	0.551511000	-0.640948000	0.843227000
B	0.622022000	0.670363000	0.311589000	B	2.063696000	-0.330410000	0.178120000
B	-3.191257000	-1.507349000	0.016291000	B	1.341565000	2.372363000	-0.119301000
B	-2.135548000	-0.333379000	-0.386418000	B	-0.092987000	-2.103144000	0.549703000
B	-1.655767000	2.378062000	0.192381000	B	1.080951000	0.941178000	0.606544000
B	0.986273000	-0.934401000	0.466888000	B	2.494968000	1.279358000	-0.441385000
B	-1.627424000	-1.989028000	0.087933000	B	-1.075804000	-0.919170000	0.896388000

**2a**				**2b**			
B	-0.395452000	-0.395247000	0.789886000	B	0.687427000	-2.103763000	-0.524216000
B	0.375188000	2.316943000	-0.837406000	B	3.688863000	1.503895000	0.091492000
B	-0.158479000	0.916187000	-1.748095000	B	-0.082173000	-0.678482000	-0.509437000
B	-0.054309000	3.737461000	1.578220000	B	2.135336000	1.977951000	-0.096445000
B	0.248661000	0.886361000	0.000000000	B	2.177437000	-2.381612000	-0.199859000
B	-0.166560000	2.397327000	-2.370089000	B	4.210682000	0.055650000	0.249580000
B	-0.395452000	-0.395247000	-0.789886000	B	1.543299000	-0.953739000	0.265911000
B	0.375188000	2.316943000	0.837406000	B	0.589346000	2.101158000	-0.490323000
B	-0.054309000	3.737461000	-1.578220000	Ag	-2.243564000	-0.059784000	0.105858000
B	-0.166560000	2.397327000	2.370089000	B	2.606373000	0.330040000	0.426712000
B	-0.158479000	0.916187000	1.748095000	B	3.317830000	-1.290261000	0.180881000
Ag	0.049273000	-2.425374000	0.000000000	B	-0.439603000	0.918607000	-0.676265000
B	0.087402000	3.966817000	0.000000000	B	0.985185000	0.631370000	0.098597000

2c

B	0.193729000	1.549854000	0.547371000
B	4.282628000	0.831050000	-0.109385000
B	0.478270000	-1.234232000	0.351749000
Ag	-2.532623000	-0.069794000	-0.073981000
B	3.320143000	2.034413000	0.076068000
B	3.889097000	-0.718562000	-0.193360000
B	2.079731000	-0.942324000	-0.327433000
B	1.628484000	-2.361497000	0.333853000
B	-0.484617000	0.108721000	0.468892000
B	2.683624000	0.623028000	-0.447096000
B	1.028882000	0.354088000	-0.181234000
B	3.141553000	-2.129988000	0.068975000
B	1.708262000	1.969484000	0.181714000

2d

B	1.833824000	-1.949970000	0.018132000
B	0.243377000	2.056819000	0.795751000
B	3.094382000	-1.389537000	-0.862146000
B	1.542197000	2.411581000	0.017349000
B	0.399570000	-2.144818000	0.712720000
Ag	-1.962951000	-0.008966000	-0.241821000
B	2.409902000	-0.265615000	0.097834000
B	-0.291455000	0.592737000	1.230747000
B	1.027106000	-0.665494000	0.957823000
B	2.677002000	1.383018000	-0.516097000
B	3.487034000	0.091247000	-1.081096000
B	1.445015000	0.943075000	0.713687000
B	-0.567665000	-1.016576000	1.230288000

2e

B	0.746748000	0.237849000	0.000000000
B	0.038907000	1.749857000	2.379211000
B	0.038907000	1.749857000	-2.379211000
B	-1.024989000	2.548404000	1.593953000
B	-1.024989000	2.548404000	-1.593953000
B	2.266693000	0.185914000	0.771486000
B	-0.325317000	1.259671000	-0.844113000
B	-1.249761000	2.557348000	0.000000000
B	-0.325317000	1.259671000	0.844113000
B	1.158097000	0.780164000	1.767626000
B	1.158097000	0.780164000	-1.767626000
B	2.266693000	0.185914000	-0.771486000
Ag	-0.396146000	-1.685448000	0.000000000

3a

Au	0.021973000	-1.827246000	0.000000000
B	-0.165650000	2.904769000	2.378127000
B	-0.129262000	1.421247000	-1.755369000
B	0.048861000	4.453736000	0.000000000
B	-0.336642000	0.107831000	-0.807014000
B	-0.080159000	4.240879000	1.584803000
B	-0.165650000	2.904769000	-2.378127000
B	0.369811000	2.835591000	0.839832000
B	-0.336642000	0.107831000	0.807014000
B	-0.129262000	1.421247000	1.755369000
B	0.369811000	2.835591000	-0.839832000
B	-0.080159000	4.240879000	-1.584803000
B	0.287766000	1.396125000	0.000000000

3b

B	0.045601000	0.132092000	-0.343707000
B	0.769229000	1.580362000	-0.455075000
B	2.300331000	1.964798000	-0.150993000
B	4.463945000	-0.737803000	0.130609000
B	2.661913000	-0.942537000	0.329237000
B	2.176003000	-2.363346000	-0.303094000
B	3.915474000	2.024015000	-0.097981000
B	4.871982000	0.812494000	0.040546000
B	1.048047000	-1.218335000	-0.259343000
B	3.287509000	0.615421000	0.431545000
B	1.606426000	0.358148000	0.230200000
B	3.701121000	-2.143169000	-0.100893000
Au	-1.927789000	-0.060049000	0.023350000

**3c**			
B	-2.614765000	-2.408896000	-0.126863000
B	0.000890000	0.887446000	0.772871000
B	-3.726094000	-1.250455000	-0.439900000
B	-1.609757000	0.592422000	0.684660000
B	-1.171582000	-2.198579000	0.431131000
Au	1.359898000	-0.164318000	-0.420627000
B	-2.202118000	-0.948316000	0.440526000
B	-1.000467000	2.091040000	0.533735000
B	-4.527697000	0.132282000	-0.762664000
B	-3.168734000	0.342261000	0.099567000
B	-3.995908000	1.577615000	-0.532453000
B	-2.542149000	2.009603000	0.093577000
B	-0.433513000	-0.769463000	0.728210000

**3d**			
Au	0.020908000	-1.920189000	0.000000000
B	0.168196000	3.763979000	2.131572000
B	0.027313000	1.040350000	-1.362254000
B	0.162658000	2.241789000	2.403530000
B	0.162658000	2.241789000	-2.403530000
B	0.218615000	4.528593000	0.791437000
B	0.168196000	3.763979000	-2.131572000
B	0.027313000	1.040350000	1.362254000
B	-0.813218000	1.487110000	0.000000000
B	-0.390961000	2.810626000	0.878069000
B	0.218615000	4.528593000	-0.791437000
B	0.111229000	0.081199000	0.000000000
B	-0.390961000	2.810626000	-0.878069000

**3e**			
B	-0.658323000	0.558270000	0.000000000
B	-0.010422000	2.157024000	-2.401632000
B	-0.010422000	2.157024000	2.401632000
B	0.984418000	3.018164000	-1.595618000
B	0.984418000	3.018164000	1.595618000
B	-2.222290000	0.637037000	-0.771935000
B	0.317960000	1.709453000	0.845009000
B	1.179924000	3.052056000	0.000000000
B	0.317960000	1.709453000	-0.845009000
B	-1.116436000	1.180485000	-1.794235000
B	-1.116436000	1.180485000	1.794235000
B	-2.222290000	0.637037000	0.771935000
Au	0.226072000	-1.330041000	0.00000000

**3f**			
B	-5.357987000	-1.026024000	0.064093000
B	-1.837826000	1.262107000	-0.099076000
B	-3.812130000	-1.420299000	-0.014878000
B	-4.960543000	1.681655000	0.105092000
B	-5.915337000	0.449575000	-0.012053000
B	-3.346305000	1.652606000	0.045621000
B	-2.193693000	-1.669705000	-0.023371000
Au	2.235677000	0.113250000	0.000997000
B	-4.287990000	0.207901000	-0.296068000
B	-1.114738000	-0.178622000	-0.072626000
B	-0.631584000	-1.833248000	0.015453000
B	-2.777088000	-0.077495000	0.255638000
B	0.531467000	-0.835926000	0.020667000

**4a**			
B	3.418818000	1.918247000	0.572012000
B	1.019947000	-0.167855000	-0.274604000
B	1.954823000	2.209424000	0.995273000
B	0.017865000	-1.370559000	0.145594000
B	3.987546000	0.557027000	-0.088459000
B	1.508943000	-1.834757000	-0.312255000
B	2.214495000	0.979826000	-0.029322000
Cu	-1.968313000	-1.149569000	0.435682000
Cu	-2.199697000	1.109671000	-0.518787000
B	3.094110000	-2.084742000	-0.426826000
B	4.191467000	-0.992272000	-0.375544000
B	0.659585000	1.295885000	0.709255000
B	-0.530946000	0.246148000	0.255770000
B	2.637802000	-0.524967000	-0.688881000

**4b**			
B	0.774373000	2.105826000	-0.952812000
Cu	-1.226883000	-0.609239000	-0.691714000
B	-0.767388000	2.107313000	-0.953847000
B	-1.538658000	-0.998450000	1.405221000
B	1.728391000	1.417894000	0.163214000
B	-0.002782000	-1.347568000	1.224804000
B	0.001888000	1.627766000	0.427742000
B	-0.825543000	0.623832000	1.541219000
B	-1.724648000	1.421859000	0.160750000
B	-2.373970000	0.249769000	1.029456000
B	2.374011000	0.243526000	1.031879000
B	1.533801000	-1.002008000	1.405526000
B	0.826218000	0.622476000	1.541745000
Cu	1.225902000	-0.610112000	-0.691889000

**4c**				**4d**			
Cu	-2.398361000	-0.000098000	0.183294000	Cu	2.836402000	0.055218000	-0.403054000
B	0.397037000	-2.328639000	-0.376297000	B	-3.629970000	-0.839795000	-1.219774000
B	0.173537000	-0.000490000	1.196096000	B	1.275512000	1.126721000	0.061824000
B	0.185362000	-1.526525000	-1.665337000	B	-2.687227000	-1.884277000	-0.620312000
B	0.397001000	2.329034000	-0.374255000	B	0.321541000	2.315978000	-0.108895000
B	0.682527000	-1.748980000	1.072482000	B	-3.719447000	0.679073000	-0.968956000
Cu	1.640475000	0.000130000	-0.409690000	B	-1.247525000	2.173830000	0.042044000
B	-0.503423000	-0.843159000	-0.139843000	B	-1.611268000	-1.711328000	0.480792000
B	0.682524000	1.748101000	1.073997000	B	-0.246562000	0.975526000	0.738174000
B	0.068105000	0.000921000	-1.992535000	B	-2.505955000	-0.234874000	-0.013921000
B	0.185398000	1.528078000	-1.664031000	B	-2.803580000	1.621994000	-0.152024000
B	1.315504000	-0.771418000	2.160677000	B	-1.223171000	-0.425931000	1.257962000
B	1.315489000	0.769602000	2.161358000	B	-1.902205000	1.011156000	1.096705000
B	-0.503328000	0.843289000	-0.139216000	Cu	0.608401000	-0.884196000	0.300705000
**4e**				**4f**			
B	-0.242695000	2.937690000	0.000000000	B	0.104640000	-2.315955000	-0.533406000
B	0.242695000	-2.937690000	0.000000000	Cu	0.589688000	0.012066000	-0.824547000
B	-0.372112000	2.265083000	1.407381000	B	1.588559000	-2.073140000	-0.078192000
B	0.372112000	-2.265083000	1.407381000	B	0.302542000	1.487245000	0.941950000
B	-0.372112000	2.265083000	-1.407381000	B	-1.135946000	-1.474071000	-1.067109000
B	0.372112000	-2.265083000	-1.407381000	B	0.598348000	0.014400000	1.544030000
B	-0.789583000	1.345374000	0.000000000	B	-1.650950000	-0.039669000	-1.362577000
B	0.789583000	-1.345374000	0.000000000	B	-0.002144000	2.312437000	-0.537207000
B	-0.372112000	0.716914000	1.664175000	B	0.365713000	-1.469531000	0.941169000
B	0.372112000	-0.716914000	-1.664175000	B	1.492885000	2.139059000	-0.085591000
B	-0.372112000	0.716914000	-1.664175000	B	1.982304000	-0.778382000	0.759495000
B	0.372112000	-0.716914000	1.664175000	B	1.945149000	0.866413000	0.756956000
Cu	1.222902000	0.707362000	0.000000000	B	-1.204893000	1.418410000	-1.073137000
Cu	-1.222902000	-0.707362000	0.000000000	Cu	-1.345931000	-0.027104000	0.788964000
**4g**				**4h**			
Cu	1.858207000	-1.213222000	-0.138453000	B	0.000000000	2.451673000	-1.148856000
B	-4.086670000	0.769152000	-0.327212000	B	-1.406743000	-1.989107000	-0.644440000
B	-1.231939000	0.848913000	-0.129084000	B	-1.406743000	1.989107000	-0.644440000
B	-2.798502000	1.739354000	-0.048563000	B	0.000000000	-2.451673000	-1.148856000
B	-1.411401000	-2.344360000	0.473967000	B	1.406743000	1.989107000	-0.644440000
Cu	1.859577000	1.212605000	-0.136051000	B	1.406743000	-1.989107000	-0.644440000
B	-1.232470000	-0.848928000	-0.127505000	B	0.000000000	1.613462000	0.332913000
B	-1.409827000	2.345262000	0.470727000	B	-1.645688000	-0.816715000	0.354708000
B	-2.799961000	-1.738357000	-0.045854000	B	-1.645688000	0.816715000	0.354708000
B	-0.086495000	-1.589461000	0.790502000	Cu	0.000000000	0.000000000	-1.173268000
B	-0.085741000	1.589494000	0.788885000	B	1.645688000	0.816715000	0.354708000
B	0.327241000	-0.000164000	0.458679000	B	1.645688000	-0.816715000	0.354708000
B	-2.660189000	0.000201000	-0.386373000	Cu	0.000000000	0.000000000	1.654442000
B	-4.087191000	-0.767530000	-0.326043000	B	0.000000000	-1.613462000	0.332913000
**4i**				**4j**			
B	0.703878000	1.430646000	1.732174000	B	1.632959000	0.000000000	0.752139000
B	-0.049784000	0.147375000	-2.293308000	B	-0.816171000	1.413565000	0.751749000
B	-0.049784000	0.147375000	2.293308000	B	-1.413565000	-0.816171000	-0.751749000
B	0.703878000	1.430646000	-1.732174000	B	-1.632959000	0.000000000	0.752139000

B	-0.915627000	-0.836649000	1.465291000	B	-0.816171000	-1.413565000	0.751749000
B	-0.915627000	-0.836649000	-1.465291000	B	1.413565000	0.816171000	-0.751749000
Cu	-0.988881000	0.941711000	0.000000000	B	0.000000000	1.632959000	-0.752139000
B	0.815606000	-0.205159000	-0.860577000	B	1.413565000	-0.816171000	-0.751749000
B	0.961539000	2.697558000	0.770377000	B	0.000000000	-1.632959000	-0.752139000
Cu	0.509547000	-2.063974000	0.000000000	B	0.816171000	1.413565000	0.751749000
B	0.815606000	-0.205159000	0.860577000	B	-1.413565000	0.816171000	-0.751749000
B	-1.414607000	-1.190095000	0.000000000	B	0.816171000	-1.413565000	0.751749000
B	0.961539000	2.697558000	-0.770377000	Cu	0.000000000	0.000000000	2.109853000
B	1.163523000	1.231676000	0.000000000	Cu	0.000000000	0.000000000	-2.109853000
**4k**				**4l**			
Cu	-1.030244000	-1.135056000	-0.400958000	B	-2.928194000	2.107504000	-0.325075000
B	1.942241000	0.612522000	1.282772000	Cu	2.329006000	-1.035507000	-0.379835000
B	-0.456266000	1.817141000	-1.881020000	B	-1.441039000	2.243138000	0.099212000
B	-2.068607000	-0.186408000	1.370469000	B	-1.848433000	-1.962721000	0.552036000
Cu	1.503432000	-0.849341000	-0.350777000	B	-3.775390000	0.744171000	-0.465575000
B	-0.111606000	1.886078000	-0.248320000	B	-0.312809000	-1.647791000	0.933104000
B	-1.202024000	1.112701000	0.746243000	Cu	1.720592000	1.208003000	0.229089000
B	-0.658877000	0.098798000	2.113667000	B	-3.452730000	-1.912711000	0.373246000
B	1.033729000	1.740082000	-1.503893000	B	-0.392017000	1.028476000	0.316603000
B	-2.541204000	0.387190000	0.015652000	B	0.583848000	-0.399250000	0.495175000
B	-1.663651000	1.287012000	-0.994402000	B	-1.934626000	0.796385000	-0.429828000
B	1.707858000	1.285274000	-0.141253000	B	-1.018348000	-0.559724000	-0.086884000
B	0.911050000	0.161975000	2.344049000	B	-4.295565000	-0.716255000	-0.137727000
B	0.362871000	1.307138000	1.256094000	B	-2.672364000	-0.721699000	-0.449956000
**4m**				**4n**			
Cu	-3.208105000	0.342206000	-0.019655000	B	-0.594141000	-1.511607000	0.566345000
B	4.494686000	1.195235000	-0.000686000	B	0.253284000	2.745704000	0.221649000
Cu	-1.153188000	-1.060542000	-0.044574000	B	0.904774000	-1.880034000	0.925009000
B	5.055961000	-0.251419000	-0.208522000	B	1.674443000	2.173544000	0.491343000
B	-1.380157000	1.015842000	0.051735000	B	-2.076423000	-0.743258000	0.476182000
B	2.915272000	1.581460000	0.032991000	B	-1.109328000	1.917605000	0.088188000
B	0.967095000	-1.084210000	0.136542000	B	0.712484000	-0.472941000	0.138896000
B	4.125497000	-1.454482000	0.057817000	B	2.220479000	0.658027000	0.592073000
B	0.219539000	0.429037000	0.138888000	B	2.153807000	-0.898824000	0.943412000
B	3.401903000	-0.015146000	-0.334814000	B	0.482861000	1.175927000	-0.115964000
B	-0.253115000	2.024153000	-0.020384000	B	-0.810618000	0.132570000	-0.048476000
B	1.342428000	1.889803000	-0.020966000	Cu	-3.902795000	-0.322088000	-0.298545000
B	2.495888000	-1.417848000	0.167635000	B	-2.292192000	0.789862000	0.203266000
B	1.910504000	0.253928000	0.372291000	Cu	3.640825000	-0.382494000	-0.474201000
**4o**				**4p**			
Cu	-3.204005000	0.369675000	0.018673000	Cu	-0.887923000	-1.782062000	0.000000000
B	4.503148000	1.254763000	0.057909000	B	-0.299520000	1.496409000	0.778536000
Cu	-1.154309000	-1.080245000	-0.007580000	B	0.116638000	0.190016000	1.702773000
B	5.044828000	-0.230111000	0.079963000	B	0.629818000	0.342483000	0.000000000
B	-1.364215000	1.021573000	-0.009755000	B	1.071290000	-1.015607000	-0.847862000
B	2.942685000	1.637282000	-0.020062000	B	0.116638000	0.190016000	-1.702773000
B	0.908820000	-1.175938000	-0.020228000	B	0.259161000	-1.297679000	-2.270832000
B	4.103481000	-1.501176000	0.080073000	B	0.635134000	-2.599012000	1.497995000
B	0.243414000	0.424776000	-0.052054000	B	0.876815000	-3.025303000	0.000000000
B	3.452468000	0.006271000	-0.164204000	B	0.259161000	-1.297679000	2.270832000

B	-0.228422000	2.056423000	0.009675000	B	1.071290000	-1.015607000	0.847862000
B	1.341333000	1.893572000	-0.006852000	B	-0.299520000	1.496409000	-0.778536000
B	2.475725000	-1.440454000	-0.010487000	B	0.635134000	-2.599012000	-1.497995000
B	1.854957000	0.174325000	-0.008321000	Cu	0.013433000	3.356987000	0.000000000
**4q**				**4r**			
B	0.203932000	2.510455000	0.000000000	B	-4.394942000	1.464188000	-0.645294000
B	-0.726912000	-2.295054000	0.000000000	Cu	2.326792000	-1.243286000	-0.226383000
B	0.768376000	0.956472000	0.876313000	B	-3.077016000	1.979674000	-0.004717000
B	-0.435967000	-1.703746000	1.393033000	B	0.303157000	-1.298744000	0.239619000
B	0.126858000	2.376203000	1.570322000	Cu	2.415515000	1.420216000	-0.028644000
B	-0.435967000	-1.703746000	-1.393033000	B	-1.248365000	-1.569226000	0.328349000
B	0.768376000	0.956472000	-0.876313000	B	-4.922481000	0.036432000	-0.769773000
Cu	-1.194143000	0.070126000	0.000000000	B	-2.145479000	-0.344408000	1.069712000
B	0.126858000	2.376203000	-1.570322000	B	-1.904578000	1.191261000	0.617837000
B	0.119440000	-0.388951000	2.077563000	B	-0.612535000	0.088999000	0.481352000
B	0.160039000	1.130566000	2.468269000	B	-4.281160000	-1.299049000	-0.323871000
B	0.119440000	-0.388951000	-2.077563000	B	1.010581000	0.119523000	0.236955000
B	0.160039000	1.130566000	-2.468269000	B	-2.883063000	-1.603185000	0.275561000
Cu	1.029571000	-0.924694000	0.000000000	B	-3.349503000	0.208340000	-0.026575000
**4s**				**4t**			
B	-2.049097000	0.449791000	0.000000000	B	-2.645720000	0.531375000	0.000000000
B	-0.154441000	-0.688171000	1.821655000	B	1.115869000	-2.024670000	0.000000000
B	-0.009574000	2.046671000	-1.487166000	B	-1.564121000	-0.635810000	0.000000000
Cu	0.869995000	0.581104000	0.000000000	B	2.080529000	-0.820402000	0.000000000
B	-1.101792000	0.721648000	-1.312489000	B	-2.312735000	2.054858000	0.000000000
B	0.336048000	0.730912000	-2.269281000	Cu	0.389767000	-0.469892000	1.423015000
B	-0.099778000	2.587295000	0.000000000	B	-1.176134000	0.964083000	-0.902525000
Cu	0.320125000	-1.779037000	0.000000000	B	-0.425501000	-1.813847000	0.000000000
B	-1.447152000	-0.855599000	-0.838769000	B	-0.798482000	2.446196000	0.000000000
B	0.336048000	0.730912000	2.269281000	B	0.389767000	1.543339000	0.844847000
B	-0.009574000	2.046671000	1.487166000	Cu	0.389767000	-0.469892000	-1.423015000
B	-0.154441000	-0.688171000	-1.821655000	B	1.601601000	0.698198000	0.000000000
B	-1.447152000	-0.855599000	0.838769000	B	0.389767000	1.543339000	-0.844847000
B	-1.101792000	0.721648000	1.312489000	B	-1.176134000	0.964083000	0.902525000
**4u**				**4v**			
B	2.303334000	2.749298000	-0.893329000	B	3.655934000	-0.678283000	-0.852824000
B	0.000542000	-1.149543000	0.617099000	B	-1.229291000	0.665617000	0.212101000
B	3.453764000	1.985140000	-0.225098000	B	2.811308000	-1.940337000	-0.646296000
Cu	-0.424705000	-2.753881000	-0.468029000	B	-1.426275000	-0.916229000	0.054384000
B	0.754480000	2.287896000	-0.896791000	B	3.255521000	0.859776000	-0.643031000
B	-0.868141000	0.197100000	0.260910000	B	-0.060998000	1.776967000	0.290345000
B	2.085064000	1.171597000	-0.566171000	B	2.234160000	-0.511596000	-0.005806000
Cu	-2.491068000	1.428877000	0.307143000	Cu	-3.101524000	0.127130000	-0.379177000
B	3.326235000	0.525797000	0.529345000	B	1.279191000	-2.108716000	-0.278271000
B	1.805180000	-0.298961000	0.210672000	B	-0.314681000	-1.990180000	-0.198736000
B	1.375984000	-1.278135000	1.478181000	B	2.600341000	2.306302000	-0.494082000
B	0.512882000	0.569252000	-0.503624000	B	1.182579000	2.691906000	-0.042635000
B	-0.658547000	1.632033000	-0.484562000	B	1.683767000	1.115521000	0.222303000
B	2.820705000	-0.706456000	1.406505000	Cu	0.399532000	-0.346224000	0.789961000
**4w**				**4x**			

B	-1.549116000	1.992326000	-0.074696000	B	-0.204965000	3.613639000	0.320580000
B	-2.663202000	0.646920000	-0.294653000	Cu	3.487287000	-1.085326000	-0.221269000
B	0.026718000	1.616899000	-0.248770000	B	-1.558601000	3.264101000	-0.342264000
Cu	1.844279000	-1.304227000	0.107516000	B	1.940044000	-0.197847000	0.604891000
B	-3.016497000	-2.081327000	0.247847000	B	1.003723000	2.574865000	0.637590000
B	-1.513105000	-2.367076000	0.000651000	Cu	-3.109198000	-1.486333000	0.161039000
Cu	2.055751000	1.232337000	0.093186000	B	-2.101878000	1.832217000	-0.648135000
B	-4.141750000	0.903386000	0.392815000	B	0.380953000	-0.619371000	0.307676000
B	0.592245000	0.060202000	-0.524629000	B	-0.295002000	2.183163000	-0.498724000
B	-0.354596000	-1.279968000	-0.320683000	B	-1.202685000	-0.853775000	0.019724000
B	-3.796288000	-0.640303000	0.247285000	B	0.860365000	1.022785000	-0.130431000
B	-3.122074000	2.085357000	0.316265000	B	-2.267308000	0.250053000	-0.488395000
B	-1.009865000	0.394127000	-0.501533000	B	1.919882000	1.313791000	1.112571000
B	-2.072644000	-0.913578000	-0.403970000	B	-0.667444000	0.531996000	-0.545745000
**4y**				**4z**			
B	-4.186879000	-1.251886000	-0.000198000	Cu	-2.932668000	-0.124964000	-0.012684000
Cu	2.854098000	-0.512873000	-0.000162000	B	3.154713000	-1.568134000	-0.245270000
B	-2.907629000	-2.122790000	0.000044000	Cu	1.103228000	1.805848000	0.170066000
B	-0.230646000	3.084462000	-0.000038000	B	2.754120000	-1.605469000	1.240007000
B	-4.213141000	0.329948000	-0.000384000	B	-1.305498000	-0.709217000	0.965671000
B	-1.573364000	2.193355000	-0.000055000	B	2.282948000	-1.049677000	-1.487936000
B	-2.722764000	-0.463237000	0.000092000	B	0.791041000	-0.052008000	-0.944755000
B	1.223405000	2.543118000	0.000166000	B	-0.972387000	-0.523679000	-0.620803000
B	-2.894202000	1.264672000	-0.000097000	B	0.286700000	-0.040801000	0.644541000
B	-0.001824000	1.423272000	0.000204000	B	1.354270000	-1.205934000	1.877550000
B	-1.380397000	-1.685737000	0.000094000	B	-0.213780000	-0.974968000	2.018246000
B	1.649170000	1.039578000	-0.000029000	B	1.078979000	-0.726613000	-2.483141000
B	-1.401487000	0.514904000	0.000113000	B	1.800018000	-0.730200000	0.221624000
Cu	0.359653000	-0.671550000	0.000177000	B	-0.400370000	-0.562425000	-2.098550000

**5a**				**5b**			
B	2.036861000	1.683157000	0.768431000	Ag	1.487507000	-0.830424000	0.036891000
B	3.175999000	0.331530000	0.808612000	B	-2.129751000	0.836603000	-1.335618000
B	0.558365000	1.331177000	0.202955000	B	-0.269875000	1.696115000	2.290692000
Ag	-1.681762000	-1.347636000	0.441551000	B	1.960257000	1.196941000	-1.198577000
B	3.980189000	-1.685308000	-1.042056000	Ag	-1.306205000	-1.076971000	0.162695000
B	2.520560000	-1.873061000	-1.529684000	B	-0.548998000	2.067220000	0.701394000
Ag	-1.550385000	1.365489000	-0.381366000	B	0.737640000	1.947407000	-0.338619000
B	4.728219000	0.873631000	0.642545000	B	0.540392000	1.292887000	-1.988072000
B	0.016487000	-0.209889000	-0.305061000	B	-1.671326000	1.348128000	1.742133000
B	1.211823000	-1.085817000	-1.020950000	B	2.195012000	1.434723000	0.315282000
B	4.539134000	-0.544984000	-0.041602000	B	1.064183000	1.791001000	1.424259000
B	3.620740000	1.909843000	0.952813000	B	-2.142552000	1.126209000	0.234682000
B	1.563388000	0.078917000	0.313321000	B	-0.977071000	0.983039000	-2.361847000
B	2.763233000	-0.964586000	-0.208584000	B	-0.795561000	1.818949000	-0.943993000

**5c**				**5d**			
B	-1.862196000	3.148044000	-0.805223000	B	-1.826543000	-1.090296000	0.968152000
Ag	0.919746000	-2.307102000	-1.164042000	Ag	0.859328000	1.557262000	-0.282760000
Ag	2.467271000	1.312263000	-0.067320000	Ag	1.170174000	-1.121402000	-0.306726000
B	-3.296324000	-1.156903000	0.457877000	B	-1.253700000	2.093374000	0.930096000
B	-0.658910000	2.068825000	-0.726287000	B	-1.768376000	0.556524000	1.178947000
B	-0.201805000	-0.692578000	-0.463822000	B	-2.404264000	1.653467000	-0.101236000
B	-3.314450000	2.955909000	-0.279613000	B	-0.418341000	-1.983162000	1.201129000
B	-4.289205000	0.037411000	0.529118000	B	-0.244805000	1.139956000	1.650899000
B	0.470053000	0.869413000	-0.538252000	B	-1.513756000	-2.561058000	0.270884000
B	-1.725807000	-1.005435000	0.168660000	B	-3.257389000	0.897893000	-1.253668000
B	-2.179704000	1.833135000	0.072927000	B	-2.597268000	-1.751193000	-0.584750000
B	-3.955687000	1.584706000	0.309055000	B	-0.143156000	-0.459104000	1.610365000
B	-2.671542000	0.342416000	0.679726000	B	-2.724525000	-0.075088000	-0.046003000
B	-1.069095000	0.555881000	0.077646000	B	-3.352192000	-0.622560000	-1.456462000
**5e**				**5f**			
B	0.586442000	1.274835000	1.077352000	Ag	-0.640259000	-3.185119000	0.000000000
B	-0.251493000	-2.886329000	0.109972000	B	-0.394167000	1.661555000	0.782181000
B	-0.929476000	1.600815000	1.429767000	B	0.228363000	0.462644000	1.734552000
B	-1.682826000	-2.351623000	0.406584000	B	0.625875000	0.622843000	0.000000000
B	2.065085000	0.518634000	0.943576000	B	1.209645000	-0.663846000	-0.839951000
B	1.111445000	-2.048591000	0.142980000	B	0.228363000	0.462644000	-1.734552000
B	-0.711217000	0.318849000	0.455328000	B	0.669360000	-0.946704000	-2.347833000
B	-2.224497000	-0.862975000	0.703206000	B	1.179078000	-2.201561000	1.556930000
B	-2.171140000	0.631804000	1.268094000	B	1.446043000	-2.438472000	0.000000000
B	-0.474448000	-1.281836000	-0.014716000	B	0.669360000	-0.946704000	2.347833000
B	0.810776000	-0.258186000	0.224552000	B	1.209645000	-0.663846000	0.839951000
Ag	3.911618000	0.315250000	-0.316921000	B	-0.394167000	1.661555000	-0.782181000
B	2.290329000	-0.958112000	0.478141000	B	1.179078000	-2.201561000	-1.556930000
Ag	-3.719014000	0.336705000	-0.441164000	Ag	-0.195537000	3.737402000	0.000000000
**5g**				**5h**			
Ag	2.436239000	0.922271000	-0.483587000	Ag	1.359996000	1.352284000	0.255344000
B	-0.061381000	0.304131000	2.269204000	B	-4.586433000	-0.856865000	0.497398000
B	0.884880000	-1.415342000	0.285942000	B	-1.738042000	-0.860427000	0.152915000
B	-0.730184000	1.213202000	1.217708000	B	-3.283419000	-1.805683000	0.235640000
B	-0.298266000	-1.009502000	-2.208330000	B	-2.060245000	2.244962000	-0.727771000
B	0.605475000	-1.126225000	2.021641000	Ag	1.546735000	-1.321176000	0.001748000
Ag	-2.343375000	-0.515174000	0.189332000	B	-1.812373000	0.827651000	0.016379000
B	0.610406000	0.208872000	0.702473000	B	-1.896186000	-2.410769000	-0.292831000
B	0.430024000	-2.099298000	-1.295145000	B	-3.400705000	1.649110000	-0.077669000
B	-0.841762000	1.082368000	-0.355607000	B	-0.741931000	1.483743000	-1.057270000
B	-0.887455000	0.341571000	-1.753439000	B	-0.626573000	-1.645414000	-0.762774000
B	0.861377000	-2.595728000	1.437016000	B	-0.258945000	-0.025758000	-0.638551000
B	0.783148000	-3.028663000	-0.038836000	B	-3.185142000	-0.041692000	0.430500000
B	0.525301000	-0.264268000	-0.911110000	B	-4.643144000	0.671293000	0.353445000
**5i**				**5j**			
B	4.459533000	-0.979965000	-0.760850000	B	-3.484207000	2.012569000	-0.618461000
B	2.300960000	1.807085000	1.467674000	Ag	1.826606000	-1.238945000	-0.317821000
B	3.068506000	-1.818137000	-0.757982000	B	-2.008871000	2.215528000	-0.178344000
Ag	-1.739034000	-1.162095000	0.227965000	B	-2.399338000	-1.890741000	0.810003000

B	1.629444000	-2.398475000	-0.391405000	B	-4.317566000	0.632774000	-0.613394000
B	0.897836000	1.142304000	1.534952000	B	-0.881940000	-1.500939000	1.197600000
B	4.690970000	0.408381000	-0.147315000	Ag	1.377346000	1.358854000	0.211211000
B	3.562218000	1.325919000	0.602068000	B	-3.997868000	-1.884954000	0.577679000
B	1.613540000	-0.773764000	-0.352060000	B	-0.960967000	1.053128000	0.222225000
B	1.902547000	0.788598000	0.269513000	B	0.007944000	-0.310959000	0.623443000
B	3.158237000	-0.068679000	-0.428801000	B	-2.480414000	0.709208000	-0.537536000
B	0.433575000	-1.687418000	0.294546000	B	-1.560160000	-0.575036000	0.010116000
Ag	-1.232523000	1.403604000	-0.433814000	B	-4.833642000	-0.779970000	-0.115894000
B	0.215267000	-0.016034000	0.604648000	B	-3.200125000	-0.807755000	-0.375304000
**5k**				**5l**			
B	-4.259328000	0.993384000	-0.918773000	B	-0.110349000	4.137436000	-0.143458000
B	-3.090653000	-0.012742000	-0.004507000	Ag	-3.621414000	-0.874834000	-0.077640000
B	-3.238536000	1.870370000	-0.153302000	B	1.242781000	3.812924000	0.534205000
Ag	0.130991000	-0.992378000	0.115776000	B	-1.994465000	0.252865000	-0.826791000
B	-0.710044000	1.072366000	0.745338000	B	-1.243534000	3.064585000	-0.591502000
B	-4.317860000	-0.529539000	-1.148986000	Ag	3.238060000	-0.904022000	-0.305212000
B	-2.360612000	1.172615000	1.081727000	B	1.861191000	2.392572000	0.742716000
B	0.850052000	1.257215000	0.172223000	B	-0.429580000	-0.113315000	-0.495358000
B	-3.458833000	-1.649088000	-0.560371000	B	0.046175000	2.649291000	0.551774000
B	-1.794532000	-0.306280000	1.232905000	B	1.148922000	-0.277475000	-0.163893000
B	-0.092576000	2.439588000	-0.032191000	B	-1.022797000	1.457399000	0.042677000
B	-2.340132000	-1.561734000	0.507529000	B	2.130275000	0.843898000	0.466818000
B	-1.669569000	2.333755000	0.071588000	B	-2.070124000	1.798338000	-1.196221000
Ag	2.639642000	0.249830000	-0.238262000	B	0.515279000	1.025504000	0.487067000
**5m**				**5n**			
B	-1.942221000	2.639193000	-1.445867000	B	-4.980247000	1.513886000	0.119219000
B	-2.277156000	-0.945493000	1.720337000	Ag	1.810377000	-1.624732000	0.139630000
B	-0.526167000	2.262372000	-0.724766000	B	-3.595849000	1.807163000	0.754027000
B	-0.721102000	-0.686752000	1.503682000	B	-0.211548000	-1.182140000	-0.640580000
B	-3.314127000	2.047927000	-1.079063000	Ag	1.769987000	1.783889000	-0.143456000
B	-3.535794000	-0.245425000	1.158469000	B	-1.749255000	-1.540850000	-0.445964000
Ag	2.560781000	0.428278000	0.149387000	B	-5.525377000	0.216255000	-0.481967000
B	-2.159914000	0.655959000	1.196093000	B	-2.557776000	-0.704897000	0.736086000
B	-2.055838000	1.725942000	-0.109187000	B	-2.348002000	0.907479000	0.886460000
B	0.498526000	0.103287000	0.874556000	B	-1.080015000	-0.050541000	0.303560000
B	0.676473000	1.516663000	0.023977000	B	-4.840640000	-1.159395000	-0.664240000
B	-0.693624000	1.337143000	0.799387000	B	0.501540000	0.150993000	-0.239465000
B	-3.603067000	0.955272000	0.091341000	B	-3.383687000	-1.610963000	-0.393808000
Ag	-0.376318000	-1.694367000	-0.480516000	B	-3.884570000	0.156934000	0.102635000
**5o**				**5p**			
Ag	2.821937000	0.562918000	-0.049229000	B	2.281870000	2.267691000	0.429642000
B	-5.086835000	1.403950000	0.014548000	Ag	-1.736324000	-1.353756000	-0.042296000
Ag	0.639700000	-1.175402000	0.026199000	Ag	-1.328616000	1.522065000	-0.083971000
B	-5.691487000	-0.036647000	0.132123000	B	3.525190000	-2.265814000	-0.003132000
B	0.797308000	1.116912000	-0.022330000	B	1.026788000	1.259479000	0.475517000
B	-3.499518000	1.746340000	0.034053000	B	0.414783000	-1.592122000	0.498624000
B	-1.625642000	-0.957508000	-0.206887000	B	3.752804000	1.905291000	0.058150000
B	-4.789262000	-1.249512000	-0.182694000	B	4.580940000	-1.145070000	-0.205720000
B	-0.821336000	0.532229000	-0.119577000	B	-0.000767000	-0.045795000	0.706063000
B	-4.034814000	0.145894000	0.298010000	B	1.930888000	-2.082012000	0.197600000

B	-0.328799000	2.106823000	0.139921000	B	2.549761000	0.829336000	-0.292062000
B	-1.920564000	2.009215000	0.123161000	B	4.348286000	0.457337000	-0.245727000
B	-3.158615000	-1.250524000	-0.267601000	B	2.989607000	-0.787288000	-0.403851000
B	-2.521452000	0.411482000	-0.367303000	B	1.410281000	-0.383136000	-0.028194000
**5q**				**5r**			
B	-0.745535000	0.179748000	0.799117000	B	3.619302000	-0.049882000	0.786531000
B	2.449534000	-0.087580000	-0.868626000	Ag	-0.849831000	-0.087709000	3.696621000
B	-2.268558000	0.059294000	0.326216000	B	3.619302000	-0.049882000	-0.786531000
B	1.512584000	-1.311768000	-1.217785000	B	-0.470277000	0.547800000	1.617926000
Ag	-4.291397000	-0.291715000	0.03166100	B	2.373657000	0.013161000	1.770239000
B	1.605206000	1.300063000	-0.922733000	Ag	-0.849831000	-0.087709000	-3.696621000
B	0.195756000	1.373330000	0.129781000	B	2.373657000	0.013161000	-1.770239000
B	0.073849000	-1.787230000	-0.872363000	B	-0.654866000	0.472983000	0.000000000
B	-1.108665000	-1.115654000	-0.092516000	B	2.207660000	-0.347636000	0.000000000
B	0.699982000	2.604006000	-0.795452000	B	-0.470277000	0.547800000	-1.617926000
B	-1.560470000	1.557648000	0.496704000	B	0.776543000	-0.117562000	0.823280000
Ag	4.180194000	-0.240949000	0.32920600	B	0.912787000	0.368274000	-2.390641000
B	0.577046000	-0.290332000	-0.243731000	B	0.912787000	0.368274000	2.390641000
B	-0.675843000	2.768094000	-0.104866000	B	0.776543000	-0.117562000	-0.823280000
**5s**				**5t**			
Ag	-2.840865000	-0.025621000	0.042262000	B	2.058807000	3.131984000	0.123480000
B	3.388535000	-2.062296000	0.336910000	B	-0.424658000	1.006728000	0.295672000
Ag	1.403544000	1.673169000	-0.234763000	B	2.194469000	0.041051000	0.548610000
B	2.991403000	-1.504835000	1.710800000	B	-0.860100000	-0.518118000	-0.376890000
B	-1.067566000	-0.702979000	1.000853000	B	-1.574301000	1.264362000	-0.936531000
B	2.508383000	-2.110080000	-1.006415000	B	0.974597000	0.837351000	1.231293000
B	1.038156000	-1.054350000	-0.919840000	B	-2.371428000	-0.059043000	-0.605693000
B	-0.724475000	-1.117192000	-0.527573000	B	0.757918000	3.429219000	-0.658652000
B	0.554883000	-0.196802000	0.456401000	B	-0.500875000	2.454747000	-0.778821000
B	1.586562000	-0.934447000	2.145859000	Ag	3.847613000	-1.008084000	-0.135653000
B	0.021020000	-0.679757000	2.082244000	B	2.290567000	1.714024000	0.827522000
B	1.316224000	-2.234218000	-2.057107000	B	0.589286000	-0.439209000	0.309431000
B	2.050322000	-1.060308000	0.418833000	B	0.663949000	2.339535000	0.544992000
B	-0.152634000	-1.829685000	-1.831458000	Ag	-4.251680000	-0.609217000	0.079864000
**5u**				**5v**			
B	2.219923000	0.494500000	0.741120000	B	-0.745535000	0.179748000	0.799117000
Ag	-1.714494000	-1.088987000	-0.164847000	B	2.449534000	-0.087580000	-0.868626000
Ag	-0.702885000	1.603278000	-0.226615000	B	-2.268558000	0.059294000	0.326216000
B	0.203222000	-2.101760000	0.858408000	B	1.512584000	-1.311768000	-1.217785000
B	1.293818000	-0.897931000	0.978958000	Ag	-4.291397000	-0.291715000	0.03166100
B	1.585276000	-2.362489000	0.022785000	B	1.605206000	1.300063000	-0.922733000
B	1.432532000	1.928387000	0.781141000	B	0.195756000	1.373330000	0.129781000
B	-0.250364000	-0.710462000	1.462378000	B	0.073849000	-1.787230000	-0.872363000
B	2.757581000	1.820180000	-0.077025000	B	-1.108665000	-1.115654000	-0.092516000
B	2.919173000	-2.265302000	-0.888817000	B	0.699982000	2.604006000	-0.795452000
B	3.515955000	0.525520000	-0.566959000	B	-1.560470000	1.557648000	0.496704000
B	0.603374000	0.704675000	1.434204000	Ag	4.180194000	-0.240949000	0.32920600
B	2.693000000	-0.989011000	0.090024000	B	0.577046000	-0.290332000	-0.243731000
B	3.749871000	-0.980649000	-1.156475000	B	-0.675843000	2.768094000	-0.104866000
**5w**				**5x**			

B	-0.646258000	-1.283512000	0.682782000	Ag	0.928719000	-0.239792000	1.412871000
B	0.223186000	2.984137000	0.151440000	B	-1.099883000	0.686991000	-2.425043000
B	0.889462000	-1.669460000	0.956534000	B	-2.502090000	-1.620546000	0.769751000
B	1.663027000	2.429830000	0.369043000	B	-0.276524000	1.741507000	1.602774000
B	-2.078558000	-0.569777000	0.445286000	Ag	0.928719000	-0.239792000	-1.412871000
B	-1.149274000	2.133542000	-0.012913000	B	-2.382940000	-0.169877000	0.000000000
B	0.710464000	-0.225135000	0.229010000	B	-1.554087000	1.026998000	0.836920000
B	2.084017000	0.901466000	0.535610000	B	-0.288856000	1.982628000	0.000000000
B	2.154542000	-0.748131000	0.825994000	B	-2.502090000	-1.620546000	-0.769751000
B	0.490116000	1.401412000	-0.142479000	B	-1.099883000	0.686991000	2.425043000
B	-0.833281000	0.379858000	-0.006581000	B	-1.961477000	-0.487281000	1.789923000
Ag	-4.103682000	-0.342117000	-0.189404000	B	-1.961477000	-0.487281000	-1.789923000
B	-2.289681000	1.029691000	0.238152000	B	-0.276524000	1.741507000	-1.602774000
Ag	3.974133000	-0.377449000	-0.265051000	B	-1.554087000	1.026998000	-0.836920000
**5y**				**5z**			
B	-1.872754000	2.904502000	-1.462300000	B	-4.296101000	1.245110000	0.009888000
B	-2.481556000	-0.662534000	1.643870000	B	-3.477166000	-0.305120000	-0.045385000
B	-0.479627000	2.164750000	-1.065909000	B	-3.308911000	2.492890000	0.004256000
B	-0.930208000	-0.477795000	1.237559000	Ag	0.384874000	-1.109265000	-0.019525000
B	-3.274597000	2.635637000	-0.852686000	B	-0.839219000	0.831907000	-0.016945000
B	-3.687701000	0.239295000	1.220714000	B	-5.125344000	-0.168791000	0.055739000
Ag	2.623684000	0.635363000	0.210215000	B	-2.548460000	1.036257000	-0.041964000
B	-2.162217000	0.814266000	1.023046000	B	0.830365000	1.145252000	-0.007407000
B	-1.949643000	2.003831000	-0.119634000	B	-4.442978000	-1.595205000	0.069467000
B	0.425235000	-0.156396000	0.346690000	B	-1.835688000	-0.570620000	-0.024731000
B	0.693657000	1.159777000	-0.568885000	B	-0.103046000	2.349062000	-0.012730000
B	-0.644764000	1.096995000	0.368991000	B	-2.862323000	-1.816558000	0.035161000
B	-3.666559000	1.528125000	0.276300000	B	-1.701142000	2.473022000	-0.014195000
Ag	-0.492754000	-2.044986000	-0.428061000	Ag	2.775766000	0.352115000	0.018338000

**6a**				**6b**			
B	-2.359528000	0.997074000	-1.379674000	B	-0.090782000	4.303030000	0.258336000
B	-3.424395000	-0.297347000	-0.886587000	Au	3.407323000	-0.657503000	-0.088699000
B	-0.867069000	0.927545000	-0.765900000	B	-1.461984000	4.001126000	-0.388089000
Au	1.421706000	-1.462028000	-0.223865000	B	1.875904000	0.411937000	0.688614000
B	-4.163997000	-1.613820000	1.535894000	B	1.059236000	3.224643000	0.642224000
B	-2.692123000	-1.593859000	2.035384000	Au	-3.266635000	-0.803119000	0.055019000
Au	0.708058000	1.720275000	0.231138000	B	-2.067407000	2.578266000	-0.657643000
B	-5.008280000	0.165776000	-0.936921000	B	0.292973000	0.045616000	0.360653000
B	-0.219936000	-0.378593000	0.274123000	B	-0.278642000	2.844763000	-0.494415000
B	-1.419408000	-0.997432000	1.253649000	B	-1.284721000	-0.150445000	0.070468000
B	-4.759833000	-0.906573000	0.212677000	B	0.840793000	1.644009000	-0.089131000
B	-3.953734000	1.069433000	-1.617645000	B	-2.324148000	1.016004000	-0.478572000
B	-1.809722000	-0.300083000	-0.342244000	B	1.920236000	1.950023000	1.137535000
B	-2.972258000	-1.152426000	0.502344000	B	-0.704316000	1.208842000	-0.517835000

6c

Au	-3.892931000	-0.207605000	-0.086278000
Au	3.715058000	-0.229758000	-0.250617000
B	-2.013595000	-0.472079000	0.862184000
B	1.679319000	2.468747000	0.491776000
B	-2.242891000	1.039925000	0.397929000
B	2.239084000	0.975221000	0.786254000
B	-0.542221000	-1.212998000	1.014804000
B	2.210499000	-0.563984000	1.294908000
B	-1.086844000	2.142800000	0.091047000
B	0.267316000	2.986819000	0.117853000
B	-0.768651000	0.322210000	0.145908000
B	0.517509000	1.381395000	0.004632000
B	0.961791000	-1.531096000	1.414591000
B	0.751569000	-0.230781000	0.451823000

6d

B	4.106999000	-0.941297000	-1.367382000
Au	-1.492280000	0.888566000	-0.713273000
B	2.734794000	-1.605918000	-1.076849000
B	2.385599000	2.057902000	1.268902000
B	4.651317000	0.501539000	-0.877847000
B	0.917643000	1.488844000	1.428168000
Au	-0.083438000	-1.604058000	0.875524000
B	3.945135000	2.418208000	1.035209000
B	1.519047000	-0.918421000	-0.269916000
B	0.029975000	0.485159000	0.549916000
B	2.867838000	-0.015657000	-0.775424000
B	1.767912000	0.756404000	0.208187000
B	4.924032000	1.772149000	0.024011000
B	3.317390000	1.444430000	-0.115954000

6e

B	-2.185604000	3.691546000	-0.705743000
B	1.641675000	0.278476000	0.708494000
B	-1.092513000	4.606456000	-0.116469000
Au	3.019827000	-0.991717000	0.047029000
B	-1.999021000	2.109477000	-0.841085000
B	0.111850000	0.448043000	0.143821000
B	-0.617311000	3.171109000	-0.731122000
Au	-2.793756000	-0.921538000	0.239010000
B	0.317460000	4.117589000	0.449081000
B	0.721928000	2.272386000	0.062246000
B	1.812434000	1.791454000	1.296453000
B	-0.459867000	1.531991000	-0.858202000
B	-1.572421000	0.544904000	-0.278313000
B	1.431572000	3.270431000	1.192855000

6f

B	4.706209000	-0.833094000	-1.066305000
B	2.679378000	0.670539000	2.276674000
B	3.255898000	-1.344501000	-1.556556000
Au	-1.160061000	-1.453651000	0.313823000
B	1.761449000	-1.850596000	-1.616622000
B	1.191823000	0.401275000	1.942185000
B	5.028484000	-0.053655000	0.214738000
B	3.952325000	0.482047000	1.313661000
B	1.831395000	-0.514021000	-0.643402000
B	2.281989000	0.399445000	0.725614000
B	3.480400000	-0.103087000	-0.304271000
B	0.535328000	-1.508427000	-0.754892000
Au	-0.653688000	1.638154000	-0.325515000
B	0.472037000	0.118443000	0.474752000

6g

Au	-1.413517000	-0.598912000	-0.072095000
B	2.143580000	1.035441000	1.252517000
B	0.042461000	2.241164000	-2.175270000
B	-1.927316000	1.123687000	1.348833000
Au	1.343023000	-0.685125000	-0.086893000
B	0.380575000	2.377186000	-0.566618000
B	-0.835170000	2.089356000	0.533755000
B	-0.493612000	1.322845000	2.108673000
B	1.496067000	1.954909000	-1.736190000
B	-2.269379000	1.502826000	-0.131706000
B	-1.249632000	2.111070000	-1.242848000
B	2.031357000	1.510246000	-0.288571000
B	1.057912000	1.052030000	2.374675000
B	0.736962000	1.967030000	1.034749000

6h

B	-1.999838000	3.424332000	-0.031856000
B	0.414812000	1.261534000	-0.381768000
B	-2.215523000	0.298945000	-0.417242000
B	0.888933000	-0.216801000	0.334887000
B	1.662671000	1.516075000	0.734095000
B	-1.054294000	1.095504000	-1.209298000
B	2.426548000	0.117463000	0.693717000
B	-0.622608000	3.735977000	0.599849000
B	0.635191000	2.758744000	0.617846000
Au	-3.791712000	-0.689844000	0.243720000
B	-2.295162000	2.011511000	-0.724112000
B	-0.575344000	-0.171363000	-0.336269000
B	-0.671424000	2.612467000	-0.582912000
Au	4.220286000	-0.658611000	0.428928000

6i

B	0.772588000	3.130278000	-1.548736000
Au	-1.358500000	-0.706758000	-0.141673000
B	-0.766144000	3.131652000	-1.548756000
B	-1.539660000	0.562003000	1.672074000

6j

Au	3.371912000	-0.246858000	-0.368727000
B	-1.618451000	-0.768141000	0.098854000
B	-0.321495000	-1.354424000	0.939789000
B	-0.630212000	0.385919000	0.676810000

B	1.727380000	2.421766000	-0.483688000	B	0.595812000	1.474925000	0.838827000
B	0.001120000	0.804665000	2.100155000	B	-0.712193000	1.966390000	-0.212981000
B	0.002891000	2.799463000	-0.112965000	B	0.618040000	2.846913000	-0.019806000
B	-0.829261000	1.965341000	1.086158000	B	2.366281000	-0.654623000	1.572486000
B	-1.722163000	2.424836000	-0.483694000	B	2.436117000	0.929588000	1.177771000
B	-2.294128000	1.306329000	0.519901000	B	1.143385000	-1.667797000	1.512306000
B	2.297268000	1.302204000	0.519884000	B	0.781461000	-0.098186000	1.369289000
B	1.541407000	0.559099000	1.671905000	B	-1.814486000	0.762107000	-0.392231000
B	0.833598000	1.963815000	1.086116000	B	1.968106000	2.376600000	0.604687000
Au	1.356925000	-0.709157000	-0.141767000	Au	-3.676492000	-0.145501000	-0.148096000
**6k**				**6l**			
Au	-2.460274000	0.102108000	0.023683000	B	5.380312000	-1.436868000	0.266462000
B	3.459681000	2.619867000	-0.405778000	Au	-1.199120000	1.745544000	0.144382000
Au	1.578256000	-1.288604000	0.203503000	B	3.995533000	-1.693521000	0.909435000
B	3.114171000	1.958370000	-1.748552000	B	0.579049000	1.146536000	-0.770590000
B	-0.881487000	0.886983000	-0.985332000	Au	-1.241229000	-1.793249000	-0.133312000
B	2.543883000	2.734846000	0.909502000	B	2.122133000	1.521796000	-0.566498000
B	1.165566000	1.594267000	0.908270000	B	5.917507000	-0.191358000	-0.448975000
B	-0.577813000	1.365386000	0.572105000	B	2.936105000	0.798371000	0.663921000
B	0.754013000	0.498323000	-0.361185000	B	2.728481000	-0.810606000	0.949166000
B	1.755746000	1.315175000	-2.196222000	B	1.468402000	0.115022000	0.341434000
B	0.226348000	0.951882000	-2.051067000	B	5.219760000	1.154991000	-0.763360000
B	1.325680000	2.889500000	1.918896000	B	-0.119442000	-0.172991000	-0.226535000
B	2.155599000	1.564446000	-0.457735000	B	3.759368000	1.610568000	-0.536590000
B	-0.090356000	2.296155000	1.768634000	B	4.281478000	-0.091612000	0.132505000
**6m**				**6n**			
B	-2.471790000	-0.074477000	0.909991000	Au	3.677697000	-0.497161000	-0.116975000
Au	1.342982000	0.935428000	-0.103602000	B	0.847612000	3.499861000	-0.223916000
Au	0.213484000	-1.494685000	-0.152798000	B	0.520448000	-0.891544000	1.389852000
B	-0.301288000	2.332231000	0.790094000	B	2.139013000	2.684207000	0.043282000
B	-1.547549000	1.301929000	1.113639000	B	1.966125000	-0.351937000	1.078980000
B	-1.625995000	2.626613000	-0.082148000	B	-0.650120000	2.956663000	-0.097268000
B	-1.731532000	-1.573915000	0.971657000	Au	-3.833612000	-0.483605000	-0.062637000
B	0.045855000	1.002473000	1.591225000	B	-2.006057000	2.179508000	0.348841000
B	-3.035373000	-1.429236000	0.134504000	B	-0.847546000	-0.230363000	0.956520000
B	-2.869605000	2.515240000	-1.115201000	B	0.732327000	1.868914000	-0.327863000
B	-3.646124000	-0.129022000	-0.560613000	B	0.632696000	0.287917000	0.250369000
B	-0.812196000	-0.369112000	1.570748000	B	2.261258000	1.123807000	0.369023000
B	-2.810440000	1.338437000	0.021820000	B	-2.274118000	0.639028000	0.550545000
B	-3.786130000	1.295111000	-1.294599000	B	-0.775321000	1.122683000	-0.097277000
**6o**				**6p**			
B	-4.499875000	1.267552000	-0.925641000	Au	-2.303164000	0.515680000	0.020720000
B	-3.341369000	0.204561000	-0.091760000	B	5.427886000	1.676632000	0.088860000
B	-3.464005000	2.093496000	-0.113690000	Au	-0.049672000	-1.056749000	-0.035545000
Au	-0.325530000	-0.847871000	0.130313000	B	6.092782000	0.274577000	-0.075909000
B	-0.948256000	1.234705000	0.736864000	B	-0.455457000	1.308393000	0.005637000
B	-4.550422000	-0.230217000	-1.274186000	B	3.838077000	1.974403000	-0.014631000
B	-2.596999000	1.316815000	1.094570000	B	2.031190000	-0.856518000	0.106572000
B	0.626214000	1.453052000	0.197729000	B	5.220351000	-0.981576000	0.153173000
B	-3.689251000	-1.391453000	-0.772589000	B	1.191882000	0.736590000	0.095615000
B	-2.147462000	-0.191969000	1.223466000	B	4.437783000	0.403561000	-0.325451000

B	-0.322162000	2.639334000	0.034373000	B	0.672386000	2.298413000	-0.149186000
B	-2.565131000	-1.391015000	0.303126000	B	2.258923000	2.204046000	-0.085648000
B	-1.900655000	2.526492000	0.148683000	B	3.587690000	-1.036477000	0.172247000
Au	2.186250000	0.244621000	-0.165816000	B	2.871318000	0.546852000	0.262962000
**6q**				**6r**			
B	-4.006495000	3.636452000	0.000000000	B	-5.020707000	-0.944342000	0.003338000
Au	3.447499000	0.193142000	0.000000000	Au	2.256931000	-0.039617000	-0.001226000
B	-2.845301000	4.656157000	0.000000000	B	-3.794877000	-1.878438000	0.003257000
B	1.569459000	0.727841000	0.000000000	B	-1.050104000	3.406566000	-0.000949000
B	-3.943813000	2.106201000	0.000000000	B	-5.005500000	0.623003000	0.002207000
Au	-2.300448000	-1.899954000	0.000000000	B	-2.358745000	2.469287000	0.000211000
B	-1.344319000	4.444441000	0.000000000	B	-3.516326000	-0.208426000	0.002135000
B	0.000000000	0.363826000	0.000000000	B	0.415222000	2.924263000	-0.001348000
B	-2.378570000	2.950610000	0.000000000	B	-3.685788000	1.535780000	0.001169000
B	0.548588000	2.013411000	0.000000000	B	-0.769730000	1.752185000	-0.000553000
B	-2.747581000	1.146614000	0.000000000	B	-2.242127000	-1.645171000	0.002199000
B	-1.612712000	-0.028759000	0.000000000	B	0.905348000	1.443598000	-0.000862000
B	-0.252751000	3.361061000	0.000000000	B	-2.198509000	0.819966000	0.001061000
B	-1.109902000	1.589781000	0.000000000	Au	-0.510690000	-0.555829000	0.000766000
**6s**							
Au	-2.296434000	0.539295000	-0.000731000				
B	5.437795000	1.748686000	0.038106000				
Au	-0.056358000	-1.085919000	0.004812000				
B	6.071244000	0.303872000	0.037770000				
B	-0.436394000	1.295237000	-0.002832000				
B	3.858620000	2.042405000	0.000672000				
B	2.007782000	-0.910818000	-0.028837000				
B	5.215842000	-1.022794000	0.028403000				
B	1.216138000	0.729639000	-0.036620000				
B	4.451861000	0.440765000	-0.097857000				
B	0.681589000	2.336879000	0.018642000				
B	2.249478000	2.221471000	0.009491000				
B	3.582678000	-1.057504000	-0.023587000				
B	2.837478000	0.508820000	-0.007827000				

**7a**				**7b**			
B	0.494681000	2.093433000	0.000000000	B	-1.377584000	-2.226339000	0.857032000
B	3.225176000	-3.837074000	0.000000000	B	1.635276000	0.780121000	-0.712163000
B	2.007667000	1.795795000	0.000000000	B	-2.494914000	-1.035897000	0.955559000
B	1.642271000	-3.827936000	0.000000000	B	0.678759000	2.023098000	-1.006233000
B	0.956792000	0.459764000	0.000000000	B	-3.213476000	0.243194000	0.230112000
B	4.111398000	-2.521087000	0.000000000	Cu	2.953514000	-0.495349000	0.005217000
Cu	-1.098807000	3.227201000	0.000000000	B	-2.298639000	1.375414000	-0.347801000
B	2.474106000	-2.400330000	0.000000000	B	0.892055000	-0.749203000	-0.516257000
B	2.740626000	0.399247000	0.000000000	Cu	-1.448809000	-0.707555000	-0.895700000
Cu	-1.856978000	-1.645318000	0.000000000	B	-0.894409000	1.894332000	-0.985896000
B	0.000000000	-1.029862000	0.000000000	B	-1.854857000	0.536584000	1.108914000
B	3.524903000	-1.037501000	0.000000000	B	0.158451000	0.498828000	-1.382286000
B	1.642652000	-1.011975000	0.000000000	B	0.047829000	-1.827547000	0.381404000
B	0.754216000	-2.475882000	0.000000000	Cu	0.146399000	1.071070000	0.895373000
Cu	-1.108782000	0.727326000	0.000000000	B	-0.854893000	-0.747949000	1.389252000

**7c**				**7d**			
Cu	-4.980086000	-0.702180000	0.193573000	B	-0.485798000	-0.819303000	1.586880000
B	1.828636000	2.135293000	0.117449000	B	0.944827000	0.012667000	-1.632526000
B	-3.055330000	-0.555189000	-0.329877000	B	-0.485798000	-0.819303000	-1.586880000
B	2.748383000	0.923991000	0.096149000	B	0.944827000	0.012667000	1.632526000
B	-3.776761000	0.887291000	-0.164850000	B	-0.508222000	0.805374000	-1.587254000
B	0.242614000	2.247626000	0.019894000	B	-0.508222000	0.805374000	1.587254000
B	-1.449814000	-0.781015000	-0.286434000	B	0.867927000	1.489604000	0.806571000
Cu	4.425479000	-0.074309000	0.159731000	B	-1.753906000	-0.023780000	-0.794532000
Cu	2.163680000	-1.083571000	-0.120419000	B	-1.753906000	-0.023780000	0.794532000
B	1.081875000	0.652933000	-0.060852000	B	0.867927000	1.489604000	-0.806571000
B	-2.179846000	0.775518000	0.070336000	B	0.908767000	-1.465209000	0.806502000
B	-1.362044000	2.288759000	-0.088696000	B	0.908767000	-1.465209000	-0.806502000
B	0.122405000	-0.771847000	-0.258319000	Cu	-1.137023000	1.950573000	0.000000000
B	-0.582973000	0.742520000	-0.325489000	Cu	2.229729000	0.031135000	0.000000000
B	-2.949770000	2.242473000	-0.140043000	Cu	-1.083602000	-1.981485000	0.000000000
**7e**				**7f**			
B	-2.640425000	-1.555146000	-0.153113000	B	-2.322134000	-2.293446000	-0.653195000
Cu	2.497684000	1.479994000	0.109144000	B	0.196853000	-2.434186000	0.542696000
B	-3.997477000	-1.179850000	0.624274000	B	-2.611186000	-0.736811000	-0.748758000
B	-0.562623000	0.618080000	-0.591203000	Cu	-1.258085000	0.620491000	0.690195000
B	-3.719340000	1.593707000	0.361426000	B	-1.880069000	2.173780000	-0.668462000
B	-1.835284000	-0.402683000	-0.980634000	B	-1.374231000	-1.488098000	0.439240000
B	-2.530478000	2.618917000	0.113078000	B	1.462335000	-1.466575000	0.814750000
Cu	2.166429000	-1.301437000	0.100046000	B	0.016702000	-0.878049000	1.164286000
B	-4.510913000	0.278009000	0.798933000	B	-0.463786000	2.510134000	-0.078750000
B	0.508031000	1.742192000	-0.025707000	B	0.714956000	1.629261000	0.405031000
Cu	-0.395940000	-1.459924000	-0.046185000	B	1.234920000	0.177492000	0.829023000
B	0.942845000	0.212351000	-0.057877000	B	-2.608340000	0.831633000	-0.966544000
B	-2.176846000	1.201845000	-0.717116000	B	-1.125683000	-3.062196000	-0.104314000
B	-3.227750000	-0.001045000	-0.196330000	Cu	3.219046000	-0.666196000	0.569973000
B	-1.005146000	2.305553000	-0.121160000	Cu	-0.379562000	1.003547000	-1.624275000
**7g**				**7h**			
B	-0.240388000	0.740252000	-1.336406000	B	-2.344313000	0.878834000	0.163786000
B	-2.030523000	-1.287988000	1.050853000	B	-0.183449000	0.090519000	1.900845000
Cu	2.883852000	-0.093020000	-0.079323000	B	-0.950903000	-0.618334000	-1.571284000
B	-0.921258000	-2.410186000	0.962391000	B	-1.338440000	1.114898000	1.374183000
B	1.192279000	1.001584000	-0.457789000	B	0.142722000	1.793516000	1.312343000
B	-2.607740000	1.556074000	-0.079790000	B	2.444301000	1.308383000	-0.214347000
B	-1.847427000	0.465783000	-1.056336000	B	2.809582000	-0.184240000	0.006764000
B	-2.671670000	0.071946000	0.442832000	B	-2.252813000	0.156226000	-1.216327000
B	0.336346000	2.242746000	-0.862592000	B	1.406674000	2.170204000	0.506108000
B	-0.347054000	-0.907725000	1.383287000	B	1.446425000	0.446215000	1.003302000
B	-1.282926000	2.066311000	-0.866954000	Cu	-1.306165000	-0.995169000	0.513971000
Cu	-0.603080000	1.093644000	0.930049000	B	0.839593000	-1.089252000	1.717603000
B	0.959025000	-0.503857000	0.340028000	B	2.165984000	-1.208360000	0.962152000
B	0.556881000	-2.045262000	0.557553000	Cu	0.869295000	-1.065185000	-0.837177000
Cu	-0.819180000	-1.314536000	-0.925463000	Cu	-0.304757000	1.260029000	-0.752782000
**7i**				**7j**			

Cu	2.335026000	-1.676405000	0.664189000	Cu	-1.222286000	-0.725057000	-0.849910000
Cu	-3.892431000	-1.375591000	-0.211109000	Cu	2.575438000	-0.275850000	-0.086475000
Cu	3.163616000	0.249439000	-0.630369000	B	-3.071232000	0.520445000	-0.408747000
B	-3.046108000	0.397896000	-0.019355000	B	0.750051000	-1.497362000	-0.329885000
B	1.253749000	-0.176528000	-0.175944000	B	-2.857616000	-0.481509000	0.767448000
B	-2.712503000	1.963039000	0.316519000	B	0.922791000	-0.095076000	-1.150864000
B	-2.059153000	3.398074000	0.400597000	B	-1.933616000	0.973406000	0.822526000
B	1.621178000	1.508502000	-0.298977000	B	-0.275475000	-1.971644000	0.721917000
B	-1.441237000	0.644549000	0.259428000	B	-1.853049000	1.409210000	-0.877354000
B	-0.332359000	-0.497623000	-0.419103000	B	1.492584000	1.414455000	-0.822571000
B	0.689546000	2.723269000	0.153611000	B	-1.693795000	-1.481586000	1.177273000
B	-0.544645000	3.741137000	0.295910000	B	-0.198163000	1.301625000	-1.031478000
B	-1.950733000	-0.652610000	-0.533719000	B	-0.828367000	2.094793000	0.516946000
B	0.193224000	1.012316000	0.355483000	Cu	0.184506000	0.207820000	1.039533000
B	-0.986982000	2.192809000	0.693827000	B	0.627469000	2.413145000	0.016529000

7k

B	1.142305000	-1.186331000	-0.162745000
B	-0.492110000	-1.015349000	0.616803000
Cu	-2.686375000	-0.603005000	0.457589000
Cu	0.959796000	0.877318000	0.156298000
B	0.039237000	-2.266559000	-0.487031000
B	-0.822506000	0.802068000	-1.955496000
Cu	2.991875000	-0.604419000	0.162745000
B	-0.401537000	2.233146000	-1.422558000
B	-0.467454000	2.760499000	0.024124000
B	-1.155921000	1.149747000	-0.258217000
B	-0.819341000	1.910976000	1.279896000
B	-0.138005000	-0.700176000	-1.253797000
B	-1.787920000	-0.356578000	-1.487370000
B	-1.420821000	-1.780925000	-0.749254000
B	-1.014646000	0.364097000	1.351179000

8a

B	-2.099833000	0.132577000	0.000000000
B	1.650051000	-5.236188000	0.000000000
B	-2.568093000	-1.324306000	0.000000000
B	2.453973000	-3.875994000	0.000000000
B	-0.880083000	-1.069821000	0.000000000
B	0.066474000	-5.323286000	0.000000000
Ag	-2.565628000	2.183145000	0.000000000
B	0.800687000	-3.854553000	0.000000000
B	-1.727162000	-2.652628000	0.000000000
Ag	2.620452000	0.263362000	0.000000000
B	0.906887000	-1.023479000	0.000000000
B	-0.907441000	-4.064606000	0.000000000
B	0.043227000	-2.411196000	0.000000000
B	1.745967000	-2.421734000	0.000000000
Ag	0.000000000	1.077452000	0.000000000

8b

B	-3.128095000	-0.916937000	0.135560000
B	1.113980000	-0.048638000	0.128106000
B	-2.733596000	-2.366651000	-0.503192000
Ag	2.105497000	1.680153000	-0.736255000
Ag	-4.241143000	0.919438000	0.282983000
Ag	3.194825000	-0.466762000	0.778656000
B	-2.003517000	-3.724485000	-0.839270000
B	1.574905000	-1.706935000	0.001952000
B	-1.539092000	-0.978038000	-0.321989000
B	-0.473396000	0.110754000	0.506480000
B	0.700075000	-2.913466000	-0.571516000
B	-0.470505000	-3.980378000	-0.824723000
B	-2.082181000	0.122978000	0.722096000
B	0.098688000	-1.226388000	-0.505323000
B	-1.013543000	-2.420412000	-0.986793000

8c

Ag	5.354324000	-0.490105000	-0.000167000
B	-1.708373000	2.366170000	-0.000500000
B	3.249429000	-0.254212000	0.000613000
B	-2.624775000	1.144878000	-0.000499000
B	3.904916000	1.283511000	0.000036000
B	-0.126204000	2.468229000	0.000074000
B	1.640064000	-0.523506000	0.000674000
Ag	-4.492424000	0.200497000	-0.000024000
Ag	-2.036177000	-1.197836000	-0.000034000
B	-0.927096000	0.858086000	0.000059000
B	2.339753000	1.102872000	0.000117000
B	1.473652000	2.681188000	0.000009000
B	0.064547000	-0.624462000	0.000642000
B	0.699405000	0.883176000	0.000696000
B	3.052889000	2.596037000	0.000198000

8d

B	3.298462000	-0.894975000	0.773049000
Ag	-2.249640000	1.749561000	0.309061000
B	4.724594000	-0.457775000	0.171833000
B	0.786108000	0.922486000	0.251718000
B	3.931008000	2.117720000	-0.615554000
B	2.107062000	0.209591000	0.930288000
B	2.594261000	2.917169000	-0.959358000
Ag	-1.769745000	-1.567433000	-0.321257000
B	5.015440000	0.956120000	-0.419759000
B	-0.332489000	1.664427000	-0.821472000
Ag	0.994641000	-1.509504000	0.149292000
B	-0.676269000	0.232897000	-0.329087000
B	2.293112000	1.684176000	0.120132000
B	3.598618000	0.690016000	0.377140000
B	1.092688000	2.435474000	-0.767635000

8e

Ag	-2.594138000	1.857208000	0.011194000
Ag	4.332587000	0.311639000	-0.227524000
Ag	-2.473509000	-1.741062000	-0.313808000
B	1.434482000	-0.357725000	0.877801000
B	-1.187428000	0.185155000	-0.582802000
B	-0.993381000	-0.324482000	2.292879000
B	-1.837886000	-0.042251000	0.983101000
B	-0.161758000	0.085983000	0.969157000
B	-0.223166000	0.011770000	-1.864708000
B	2.151229000	-1.023845000	2.173195000
B	1.284510000	-0.380845000	-1.897466000
B	0.581230000	-0.632572000	2.390578000
B	0.645135000	0.166804000	-0.495423000
B	2.914273000	-1.060662000	0.806867000
B	2.302324000	-0.648512000	-0.669874000

8f

B	0.494651000	-0.952891000	-0.196235000
B	-0.719664000	-0.667945000	0.853560000
Ag	-2.896880000	-0.920469000	-0.167247000
Ag	1.656501000	1.198546000	-0.495169000
B	-0.561965000	-1.069234000	-1.363826000
B	-0.660218000	2.284809000	-0.642778000
Ag	2.502732000	-1.319129000	0.391331000
B	-0.707248000	3.033112000	0.738994000
B	-1.320920000	2.652101000	2.093003000
B	-1.130822000	1.337703000	0.991169000
B	-1.913968000	1.292351000	2.523530000
B	-0.492492000	0.545240000	-0.609336000
B	-1.451941000	1.479091000	-1.770936000
B	-1.626741000	-0.059594000	-1.927324000
B	-1.774791000	-0.088864000	1.858377000

8g

Ag	1.825116000	1.402244000	-0.520643000
B	-3.753457000	2.572188000	-0.416073000
B	0.589179000	-0.338926000	0.907484000
Ag	-2.086590000	-2.156014000	-0.336495000
B	0.230026000	2.771632000	0.146908000
B	-3.387716000	1.115308000	0.166398000
Ag	2.294658000	-1.366480000	0.280391000
B	-2.738261000	3.732158000	-0.504214000
B	0.094979000	1.208009000	0.950811000
B	-2.619244000	-0.251427000	0.462420000
B	-1.002075000	-0.429015000	0.777718000
B	-2.495616000	2.547669000	0.613688000
B	-1.198477000	3.572891000	0.003417000
B	-1.875843000	1.023219000	1.105284000
B	-0.955419000	2.406631000	1.207576000

**9a**				**9b**			
B	1.466282000	1.546842000	-0.066689000	Au	-5.082855000	-0.068663000	-0.121032000
B	-4.911091000	3.195351000	0.125464000	B	1.756776000	2.973400000	-0.228598000
B	0.829539000	2.937356000	-0.182036000	B	-3.025563000	0.057722000	-0.261355000
B	-4.657896000	1.643296000	0.225375000	B	2.738262000	1.799654000	-0.106186000
B	-0.282833000	1.653462000	-0.036665000	B	-3.793473000	1.589130000	-0.368159000
B	-3.757304000	4.258581000	-0.075799000	B	0.180359000	2.909880000	-0.292981000
Au	3.055376000	0.291198000	0.005470000	B	-1.440322000	-0.093945000	-0.163312000
B	-3.364424000	2.673036000	0.117755000	Au	4.283321000	0.599024000	0.045906000
B	-0.668925000	3.383527000	-0.178929000	Au	1.383815000	-1.726378000	-0.061506000
Au	-1.933020000	-1.679905000	-0.050860000	B	1.059359000	1.408589000	-0.160290000
B	-1.670342000	0.397026000	0.063356000	B	-2.210847000	1.466635000	-0.228946000
B	-2.209762000	3.911728000	-0.151708000	B	-1.439419000	3.087982000	-0.345910000
B	-1.876203000	2.031229000	-0.061287000	B	0.189372000	-0.132326000	-0.115551000
B	-3.188637000	0.976100000	0.181832000	B	-0.551423000	1.327350000	-0.216569000
Au	0.415087000	-0.421897000	0.047879000	B	-2.986937000	2.938637000	-0.391507000
**9c**				**9d**			
B	-3.105774000	1.234270000	0.255788000	B	-3.311599000	1.074379000	-0.532680000
B	1.060093000	-0.060787000	0.090520000	Au	2.937059000	0.674428000	-0.272892000
B	-2.610886000	2.406380000	1.273004000	B	-4.244458000	2.323558000	-0.128847000
Au	1.920896000	-1.823267000	0.605548000	B	-0.025620000	1.465668000	-0.174637000
Au	-4.223753000	-0.403144000	-0.402920000	B	-2.152804000	4.073477000	0.667090000
Au	2.881428000	0.987416000	-0.847805000	B	-1.586240000	1.339464000	-0.576780000
B	-1.781105000	3.560974000	1.963651000	B	-0.590983000	4.114161000	0.990162000
B	1.670350000	1.524111000	0.685962000	Au	0.313865000	-1.768967000	0.359471000
B	-1.524170000	1.019860000	0.699633000	B	-3.689730000	3.671903000	0.408088000
B	-0.546716000	0.055151000	-0.329271000	B	1.371480000	1.578126000	0.846178000
B	0.842345000	2.625054000	1.496951000	Au	-2.207295000	-0.678513000	-0.256459000
B	-0.235551000	3.689434000	2.019390000	B	0.932905000	0.142568000	0.372847000
B	-2.142886000	0.223027000	-0.565387000	B	-1.048130000	2.720717000	0.220740000
B	0.119594000	1.094490000	0.896458000	B	-2.637837000	2.561870000	-0.212194000
B	-0.886714000	2.204161000	1.707092000	B	0.493676000	2.948332000	0.804136000
**9e**				**9f**			
Au	-2.429031000	1.684188000	-0.007511000	B	-0.096096000	0.146914000	0.413146000
Au	4.434835000	0.149822000	-0.166056000	B	1.811546000	0.719327000	0.234148000
Au	-2.529360000	-1.559145000	-0.197517000	B	0.419692000	1.783787000	0.269604000
B	1.544882000	-0.362503000	0.952860000	Au	1.347134000	-0.910077000	1.541495000
B	-1.050754000	0.114696000	-0.593631000	B	-2.813155000	1.119592000	0.269481000
B	-0.895275000	-0.289639000	2.377213000	Au	2.707066000	-0.054462000	-1.409491000
B	-1.790855000	-0.065051000	1.088295000	B	-0.376609000	4.412575000	1.042018000
B	-0.094489000	0.008900000	0.991828000	B	-1.710360000	-0.080747000	0.312784000
B	-0.056490000	-0.113011000	-1.853541000	B	-1.876961000	4.111455000	0.795325000
B	2.246016000	-0.935359000	2.306485000	B	-2.519315000	2.708908000	0.450624000
B	1.442825000	-0.507368000	-1.826407000	Au	-3.654835000	-0.616540000	-0.506542000
B	0.665574000	-0.592188000	2.469273000	B	-1.187342000	1.425298000	0.106146000
B	0.752353000	0.065832000	-0.452527000	B	0.890233000	3.404730000	0.898885000
B	3.057081000	-1.002362000	0.969709000	B	1.922141000	2.218883000	0.830031000
B	2.451324000	-0.664817000	-0.566416000	B	-0.773740000	3.010326000	0.295520000
**9g**				**9h**			

Au	3.495253000	0.424037000	-0.375557000	B	2.444971000	1.078091000	0.109817000
B	-0.512508000	4.639507000	0.195516000	Au	-5.381126000	-0.309308000	0.043387000
B	-0.320539000	-0.767634000	0.610806000	B	1.755543000	2.501143000	-0.011084000
Au	-3.378538000	0.579324000	-0.385938000	B	-3.515593000	0.271411000	-0.092329000
B	2.083136000	1.507772000	0.477623000	Au	4.152674000	0.016378000	0.067625000
B	-1.501279000	3.397480000	0.487358000	B	-1.948971000	-0.068018000	0.041463000
Au	-0.113744000	-2.697205000	0.240178000	B	0.967661000	3.917994000	-0.156262000
B	1.030646000	4.555582000	0.266139000	B	-0.344979000	-0.294027000	-0.162592000
B	0.601672000	0.540577000	0.865826000	B	-0.574978000	4.101867000	-0.104570000
B	-2.179760000	1.949333000	0.503755000	B	-2.567303000	1.598417000	-0.258977000
B	-1.365873000	0.436285000	0.594307000	B	0.046476000	2.620943000	0.201642000
B	0.136481000	3.409206000	1.031374000	B	-0.926848000	1.256728000	0.036254000
B	1.785237000	3.168992000	0.605603000	B	0.749908000	1.062172000	-0.067545000
B	-0.714640000	1.920391000	1.150953000	Au	1.581064000	-1.035854000	-0.076879000
B	0.910484000	2.005231000	1.447548000	B	-1.657152000	2.948067000	-0.075131000
